# Author Correction: Modular RNA motifs for orthogonal phase separated compartments

**DOI:** 10.1038/s41467-025-61207-0

**Published:** 2025-06-26

**Authors:** Jaimie Marie Stewart, Shiyi Li, Anli A. Tang, Melissa Ann Klocke, Martin Vincent Gobry, Giacomo Fabrini, Lorenzo Di Michele, Paul W. K. Rothemund, Elisa Franco

**Affiliations:** 1 Department of Computing and Mathematical Sciences, California Institute of Technology, Pasadena, CA USA; 2 Department of Bioengineering, University of California, Los Angeles, CA USA; 3 Department of Mechanical and Aerospace Engineering, University of California, Los Angeles, CA USA; 4 Interdisciplinary Nanoscience Center (iNANO), Aarhus University, Aarhus, Denmark; 5 Department of Chemical Engineering and Biotechnology, University of Cambridge, Cambridge, UK; 6 Department of Chemistry, Molecular Sciences Research Hub, Imperial College London, London, UK; 7 fabriCELL, Molecular Sciences Research Hub, Imperial College London, London, UK; 8 Department of Bioengineering, California Institute of Technology, Pasadena, USA; 9 Department of Computation & Neural Systems, California Institute of Technology, Pasadena, USA

**Keywords:** DNA and RNA, Synthetic biology, RNA nanotechnology, Fluorescence imaging

Correction to: *Nature Communications* 10.1038/s41467-024-50003-x, published online 30 July 2024

In the version of the article initially published, the equation in the caption to Fig. 3g and Supplementary Note 2 was $A[1-{e}^{(-t/\tau )}]$ and has now been corrected to $1+A{e}^{(-t/\tau )}$ in the HTML and PDF versions of the article.

