# Supplementary material for: Modular RNA motifs for orthogonal phase separated compartments
Source: Nat Commun. 2024 Jul 30;15:6244. doi: 10.1038/s41467-024-50003-x (PMC11289419; doi:10.1038/s41467-024-50003-x)
Supplement: Supplementary file 1 — Supplementary Information file [file 41467_2024_50003_MOESM1_ESM.pdf]

Supplementary
Materials and Methods, Figures, and Tables
Modular RNA motifs for orthogonal phase separated compartments

Jaimie Marie Stewart[1,†], Shiyi Li[2,†], Anli A. Tang[3,†], Melissa Ann Klocke[3], Martin Vincent Gobry[4], Giacomo Fabrini[5,6,7], Lorenzo Di Michele[5,6,7], Paul W.K. Rothemund[1,*] and Elisa Franco[2,3,*]

[1] Department of Computing and Mathematical Sciences, California Institute of Technology; Pasadena, 91125, CA, USA

[2] Department of Bioengineering, University of California at Los Angeles; Los Angeles, CA 90024, USA

[3] Department of Mechanical and Aerospace Engineering, University of California at Los Angeles; Los Angeles, CA 90024, USA

[4] Interdisciplinary Nanoscience Center (iNANO), Aarhus University; Aarhus, DK-8000, Denmark

[5] Department of Chemistry, Molecular Sciences Research Hub, Imperial College London; London W12 0BZ, UK

[6] Department of Chemical Engineering and Biotechnology, University of Cambridge; Cambridge CB3 0AS, UK

[7] fabriCELL, Molecular Sciences Research Hub, Imperial College London; London W12 0BZ, UK

† These authors contributed equally

*Corresponding authors. Email: efranco@seas.ucla.edu, pwkr@dna.caltech.edu

1 Supplementary Notes 1
Supplementary Note 1 1
Supplementary Note 2 3
2 Supplementary Figures 6
3 Supplementary Tables 28
References cited 29

1 Supplementary Notes

Supplementary Note 1

The RNA strands were designed and optimized using the NUPACK Design tool with the script shown below[1]. A target secondary structure was denoted by DU+ notation. Sticky ends or

kissing loops were pre-selected and noted as a design restraint as well as certain repeat nucleotide sequences. We used the default energy parameters from Serra and Turner, 1995 in 1M Na+ [1].

NUPACK script for multi-stranded motif strand design:

```
-----------------------------------------------------------------------------------------------------------
material = rna
temperature[C] = 25.0    # optional units: C (default) or K
trials = 3
sodium[M] = 1.0    # optional units: M (default), mM, uM, nM, pM
dangles = some


# target structure using DU+ notation

#5' SE
structure msmotif = U7 D15 (U2 D15 (+ U7) U2 D15 (+ U7) U2 D15 (+ U7) U2)

# sequence domains
domain A2 = AA

#input sticky-end sequence
domain SE = GCUAGCA
domain S4 = N15
domain S3 = N15
domain S2 = N15
domain S1 = N15


# thread sequence domains onto target structures

#5' SE
msmotif.seq = SE S1 A2 S2 SE S2* A2 S3 SE S3* A2 S4 SE S4* A2 S1*

# specify stop conditions for normalized ensemble defect
# default: 1.0 (percent) for each target structure

msmotif.stop = 1.0

# prevent sequence patterns
prevent = AAAA, CCCC, GGGG, UUUU, KKKKKK, MMMMMM, RRRRRR, SSSSSS,
WWWWWW, YYYYYY
```

NUPACK script for single-stranded motif stem design:

```
-----------------------------------------------------------------------------------------------------------
material = rna1999
temperature = 37
trials = 10
```

structure NS= U1D20 U9 U2 D20 U9 U2 D20 U9

domain arm1= N20AAGCGCGCAN20AA
domain arm2= N20AAGCGCGCAN20AA
domain arm3= N20AAGCGCGCAN20

NS.seq = arm1 arm2 arm3

prevent = AAAA, CCCC, GGGG, UUUU, KKKKKK, MMMMMM, RRRRRR, SSSSSS, WWWWWW, YYYYYY

Supplementary Note 2

Pre-processing

Images affected by strong background noise making it impossible to process, and images that are defocused, or have overlap FOV were discarded.
All multi-stranded nanostar images for quantification were processed in FIJI (ImageJ) using a custom macro implementing the following pipeline: Images were thresholded using a custom macro implementing the following pipeline: contrast enhancement (5% pixels saturated), background subtraction with white top-hat morphological filters (element=disk, radius=20), denoising via Gaussian Blur (sigma=2), and background subtraction with a rolling ball radius of 25-50 pixels.
For single-stranded nanostars, all black-and-white fluorescence images in figures were processed in FIJI (ImageJ) using a custom macro implementing the following pipeline: calibration, contrast enhancement (10% pixels saturated), denoising via Gaussian Blur (sigma = 1.5), and background subtraction with rolling ball method (radius = 1, for extremely large droplets radius = 10). Images for peptide recruitment were thresholded with LUT window (5000, 10000) (16-bit) for the brightfield channel and at (10000,65535) for 6FAP-tagged peptides. Brightfield channel images were inverted to a black background before merging.

Segmentation

In peptide recruitment experiments using multi-stranded nanostars, we used the MaxEntropy method for thresholding. Masks were generated from the CY3 channel for the Streptavidin-binding motif, the CY5 channel for the P22-binding motif, and FITC channel images for TAT-binding motifs. For single-stranded nanostars, we used the Moment method for thresholding. All masks were saved as TIFF files for further analysis.

Chord Length Distribution (CLD) analysis

All chords mentioned in the paper are applied and calculated by functions from the Python package Porespy, particularly Porespy.filters.apply_chords, using the built-in method. In short, chords are applied as lines with fixed space (in our case, 1 pixel) within the area of interest in both the x and y directions. Objects touching the edges of the FOV are excluded (trim_edges = False). Chord lengths are then calculated as the number of pixels of each line and calibrated to micrometers.

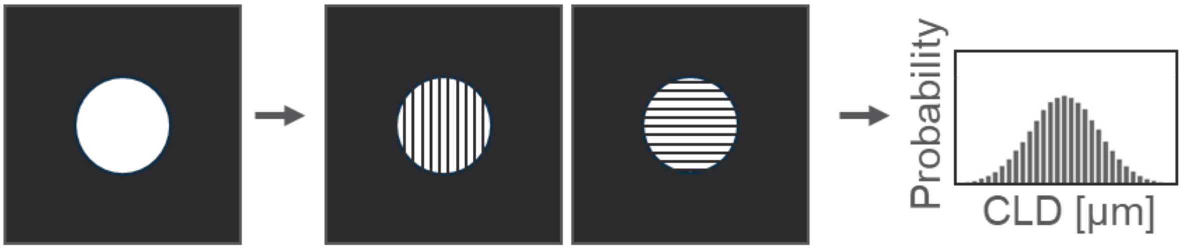

The resulting Numpy arrays are saved in NPY format via Numpy. Each sample's resulting chord lengths, along x and y and across all FOVs, are pooled together, and saved into new arrays. The reported means are calculated as mean(mean_sample1, mean_sample2, mean_sample3).

Condensate number analysis
Objects smaller than 6 px^2 (about 0.3 μm^2) were considered as noise and excluded. Droplet numbers were autogenerated by FIJI - Measure and recorded.

Coordinate angle mapping
To determine whether our nanostars including distinct fluorogenic aptamers produce condensates that mix or do not mix, we built pixel intensity histograms from fluorescence microscopy images (Fig. 4). This was done because upon DFHO staining, both Corn and Red Broccoli aptamers can be detected in the FITC channel and the Cy3 channel, albeit with varying intensity, since Corn aptamer Em_{max} = 545 nm, and Red Broccoli Em_{max} = 582 nm[2]. Images were processed to obtain the intensity of each pixel within segmented regions of interest in each channel (FITC and Cy3) and computing the angle of their coordinates in a plane with an x-axis corresponding to the CY3 channel intensity and with a y-axis corresponding to the FITC channel intensity, as sketched in Supplementary Figure 22. A line was drawn from the projected point to the origin, and the angle between this line and the x-axis was referred to as the coordinate angle. Finally, we generated a histogram based on the coordinate angles calculated from every pixel within the regions of interest.
It is important to note that although both Corn and Red Broccoli rely on the formation of G-quadruplexes to bind their fluorophore, Red Broccoli forms an intramolecular core-binding region, while Corn forms homodimer where fluorophores bind dimer interfaces[2,9]. This aptamer-directed dimerization is present in addition to designed KL-directed interactions, and results in the formation of structures other than spherical droplets for Corn-appended nanostars (Supplementary Figure 23, indicated by white arrow). These structures can be distinguished based on their non-spherical shape and were excluded from histogram generation according to their region labels.
As a control, we prepared separate samples including condensates from nanostars including Corn or Red Broccoli individually. The expectation is that if the nanostars are orthogonal, the histogram peaks measured when Corn and Red Broccoli condensates are produced in the same sample would be aligned with those of the separately annealed controls. If any mixing occurs due to the nanostar interactions, then a shift of the peaks (indicating mixing with a specific stoichiometry) or a uniform distribution (indicating mixing following a random stoichiometry) would be observed.
Subsequent processing was performed using a Python3 script. For each FOV, we generated a mask by applying a Gaussian blur (sigma = 5) and gamma enhancement (gamma = 0.5) to the CY3 channel image, thresholding it using the Otsu method, skeletonizing it to generate seeds, and growing it using a watershed transformation. For each pixel within the masks, pixel intensities for CY3, FITC channels, and the region labels were extracted into three columns and

saved in a .csv file. We manually excluded regions with unfocused droplets and non-spherical, cloudy condensates. Finally, we used a Python script to read and concatenate the data of 10 FOV into a single .csv file. The calculation of coordinate angles and histogram plotting is described below (Data visualization).

Data visualization

Violin plots were generated using a Python3 script based on Seaborn. For three NPY files storing chord length information of three repeats, the script loads the files, calibrates chord length into μm, and pools them into one array as the input dataset for violin plots. Cut = 0 was applied to ensure that the kernel density estimation doesn't exceed the data range. As for kernel density estimation for the violin plots, data was normalized to have the same area of the violins (scale = 'area'). The coordinate angle histogram was generated using a Matlab script. The script loaded .csv files in which we recorded the pixel intensity of FITC and Cy3 channels of regions of interest (condensates). The background was eliminated by subtracting the smallest value of each channel. Each row of the resulting array contained the background-subtracted pixel intensities of both channels for each pixel within the droplet region. The coordinate angle for each pixel was calculated as arctan (FITC intensity/Cy3 intensity). Histograms were normalized to have the unitary area.

Data processing for FRAP

Images are first processed to eliminate the influence of horizontal drifting using the SIFT algorithm (https://imagej.net/plugins/linear-stack-alignment-with-sift) with default parameters and no interpolation. Then, a spherical ROI was drawn at the bleached area and an ROI of the same size was generated on the surrounding unbleached droplets and used as controls. Normalized pixel intensity within both ROIs was exported and recovery was calculated as

$$(I_{bleach,t} / I_{bleach,max}) / (I_{unbleach,t} / I_{unbleach,max}) \quad (1)$$

where *I* denote mean pixel intensity among the area, *t* denotes a certain time point, *max* denotes the highest pixel intensity within the area among all time points.

Data processing for time-dependent coalescence

Images were stacked and aligned using the Registration plugin - Linear stack alignment with SIFT to correct horizontal drifting. Then images are enhanced with saturation = 5% and background subtracted with rolling ball radius = 9 for display. Seven fusion events were found and manually cropped from the epifluorescence images, exported as TIFF files, and imported into Python. For each fusion event, condensates were segmented using Otsu unsupervised thresholding as implemented in skimage.filters.threshold_otsu. The binarized image was then labeled using skimage.measure.label, which associates a different label to each segmented object. Associated geometrical properties, including centroid position, major and minor axis lengths, and orientation, were extracted via skimage.measure.regionprops_table. The Characteristic length (lc) of condensates at the initial frame was measured manually in ImageJ using the ROI tool and calculated as the mean of the two droplets' radii. For time points following contact, the Aspect Ratio was computed as the ratio between the major and the minor axis length of the best-fit ellipse and appended to a list. Time constants τ were obtained by fitting the Aspect Ratio vs time profiles with a single exponential decay with the formula $1 + Ae^{(-t/\tau)}$ using scipy.optimize.curve_fit, where a and τc are fitting parameters. Finally, computed time constants were linearly fitted against the pre-fusion characteristic droplet size (Characteristic Length, lc) using scipy.stats.linregress, yielding the inverse capillary velocity as the fit slope. Linear regression parameters (mean ± standard error), $R^{2,}$ and p-value with respect to the null hypothesis (H_0) of null slope are as follows: slope = 3468.64±1892.93, intercept: 2228.90±6195.51, R^2 = 0.63, p-value = 0.126.

2 Supplementary Figures

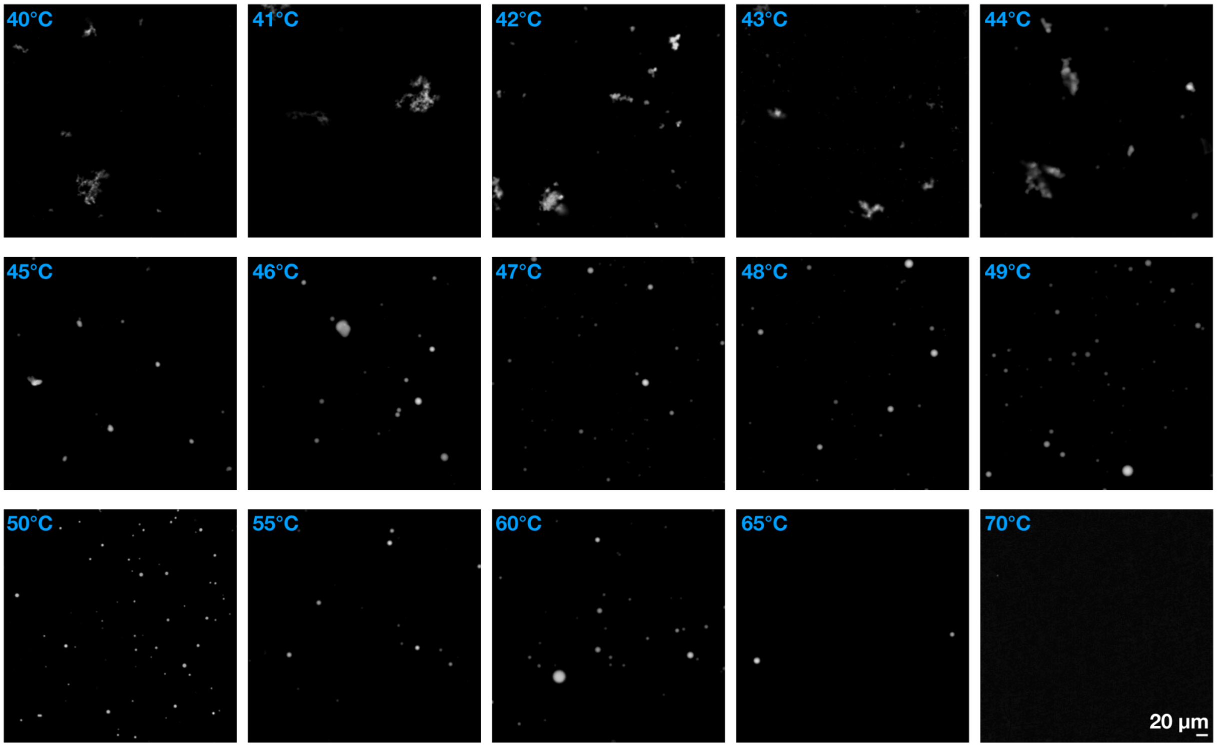

Supplementary Figure 1. 12-hour incubation over variable temperatures of multi-stranded condensates. Multi-stranded condensates (4m5) were annealed with our annealing protocol that includes a melt phase (70°C for 10 min) and hold phase (temperature between 40°C-70°C 12h) protocol. Strands were annealed in our assembly buffer (40 mM HEPES/100KCl/500 mM NaCl). Samples were stained with SYBR Gold and imaged. Experiments were repeated three times, n=3; here we provide representative images.

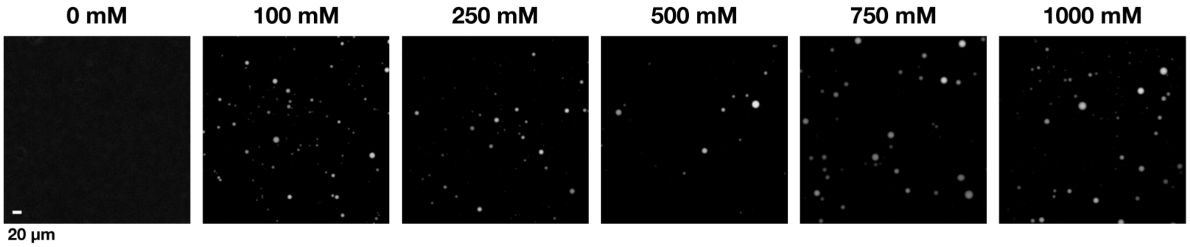

Supplementary Figure 2. Potassium titrations of multi-stranded condensates. Nanostars (4m6) were assembled in 40 mM HEPES and a variable KCl concentration. All samples underwent a thermal treatment of a 70°C denaturing steep for 10 minutes, then incubated at 50°C for 12 hours, then a quick cool to room temperature. Liquid-like condensate formation begins at 100 mM KCl and maintains droplet-like morphology through 1000 mM KCl. Samples

were stained with SYBR Gold and imaged. Experiments were repeated three times, n=3; here we provide representative images.

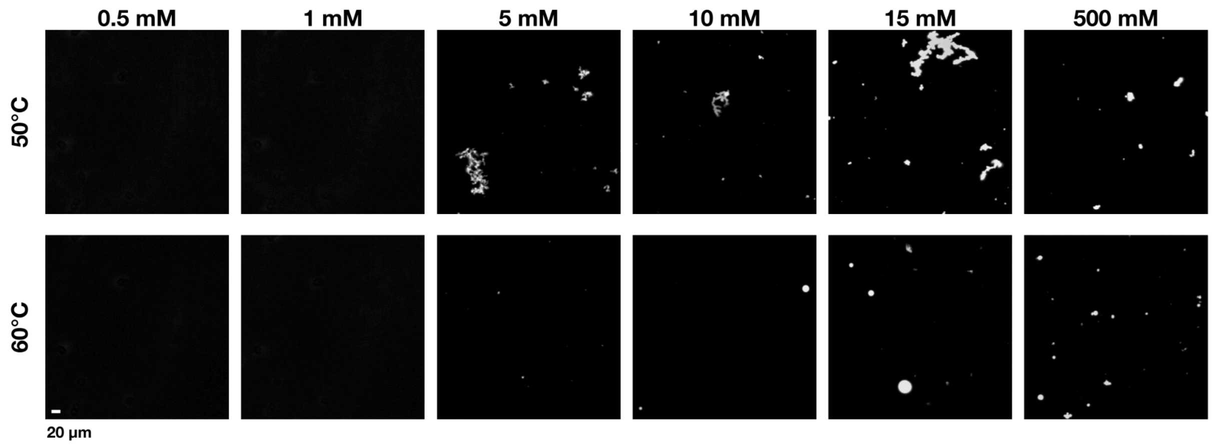

Supplementary Figure 3. Magnesium titrations of multi-stranded condensates. Nanostars (4m6) were assembled in 40 mM HEPES and a variable Magnesium concentration. Under the protocol using equimolar purified RNA strands that form an RNA motif via thermal treatment of a 70°C denaturing step for 10 minutes, then incubating at 50°C for 12 hours, then a quick cool to room temperature, the addition of Magnesium causes gel formation, starting at 5 mM. As expected, the addition of Magnesium changes the melting temperature of the condensates, in which we observe droplet-like assemblies when we raise to a 12-hour incubation step from 50°C to 60°C around 15 mM Magnesium. Samples were stained with SYBR Gold for imaging. Experiments were repeated three times, n=3; here we provide representative images.

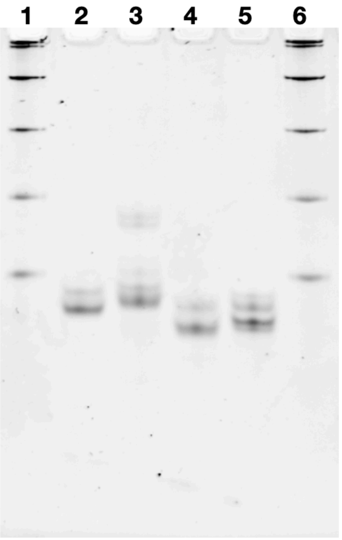

1. **Low range ssRNA Ladder**
2. **S1**
3. **S2**
4. **S3**
5. **S4**
6. **Low range ssRNA Ladder**

Supplementary Figure 4. 10% denaturing polyacrylamide gel of RNA strands S1, S2, S3,

and S4 from design 4m6 with sticky-end sequence 5'-GCUAGC. RNA was produced from in vitro T7 transcription and purified by Amicon 10K 0.5 mL centrifugal filters (as mentioned in Supplementary Section 1.2.1). Experiments were repeated three times, n=3. Target strand sizes are 39 NT, however, shorter and longer products are present which may contribute to the unwanted formation of assemblies.

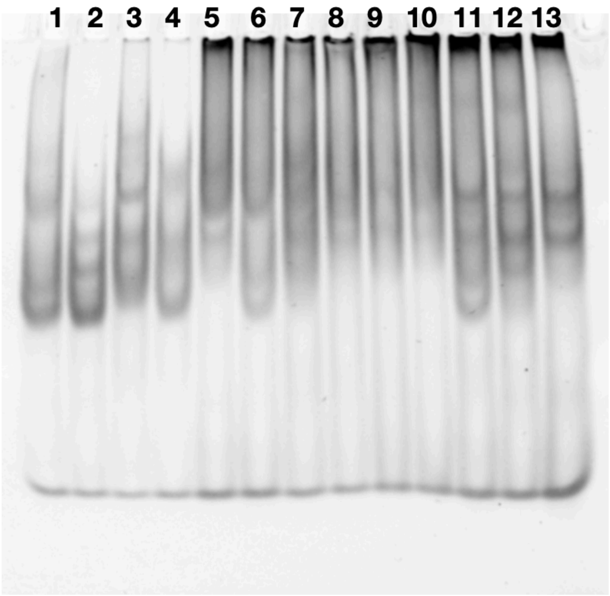

1. S1
2. S2
3. S3
4. S4
5. S1+S2
6. S1+S4
7. S2+S3
8. S3+S4
9. S1+S2+S3
10. S1+S2+S4
11. S1+S3+S4
12. S2+S3+S4
13. S1+S2+S3+S4

Supplementary Figure 5. 12% non-denaturing polyacrylamide gel of RNA strands S1, S2, S3, and S4 from design 4m6 with sticky-end sequence 5'-GCUAGC. RNA was produced from in vitro T7 transcription and purified by Amicon 10K 0.5 mL centrifugal filters (as mentioned in Supplementary Section 1.2.1). Samples were annealed either individually (lanes 1-4) or in equimolar concentrations (lanes 5-13) using the protocol in Supplementary Section 1.31. Experiments were replicated twice, n = 2. Individual strands (lanes 1-4) form secondary structures as indicated by multiple bands. Two interacting strands (lanes 5-8) annealed together form large structures as evidenced by large complexes that are present in the wells and smears. S1+S2 produces the largest amount of sample stuck in the well for two interacting strands. Three interacting strands (lanes 9-12) and the complete NS sample (lane 13) present greater prominent bands in comparison to the two-strand samples. Samples that are present in the wells may occur due to palindromic sequences that make it possible for long chains to form. Smears may also be present due to erroneous transcription products that form unwanted assemblies.

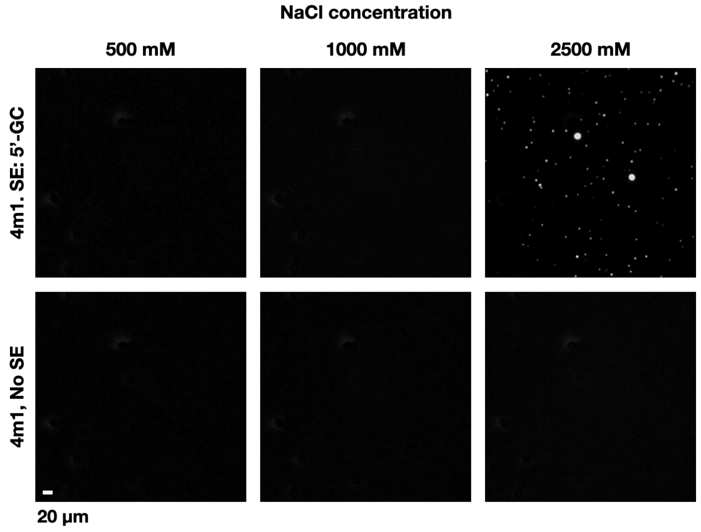

Supplementary Figure 6. NaCl titrations using multi-stranded nanostars with and without 2nt sticky ends. Nanostar with 2nt 5'-GC (4m1) required 40 mM HEPES/100 mM KCl and increased NaCl concentration to form via a thermal treatment of a 70°C denaturing step for 10 minutes, then incubating at 50°C for 12 hours. RNA nanostars without sticky-ends (4m1) do not form in comparable buffer and temperature conditions. Samples were stained and imaged with SYBR Gold. Experiments were repeated three times, n=3; here we provide representative images.

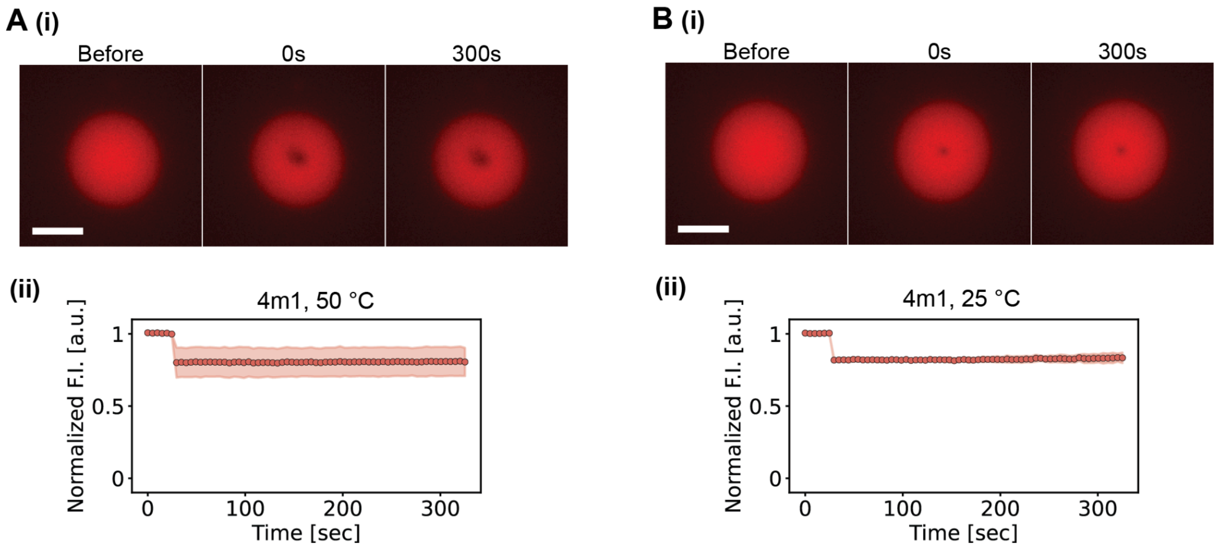

Supplementary Figure 7. Fluorescence recovery after photobleaching (FRAP) analysis of condensate formed from multi-stranded nanostar treated with melt-and-hold protocol showed little recovery. RNA was transcribed with 1% of CY3 tagged UTP and purified with size exclusion columns before imaging. Condensates were annealed with the heal-and-hold protocol and imaged at 50°C (for **A**) or at room temperature (for **B**). Orange dots indicate mean intensity at the corresponding time point. Shaded areas indicate standard error. Mean and standard error

were calculated from 3 FOVs each belonging to one replicate. Scale bar, 10 μm.

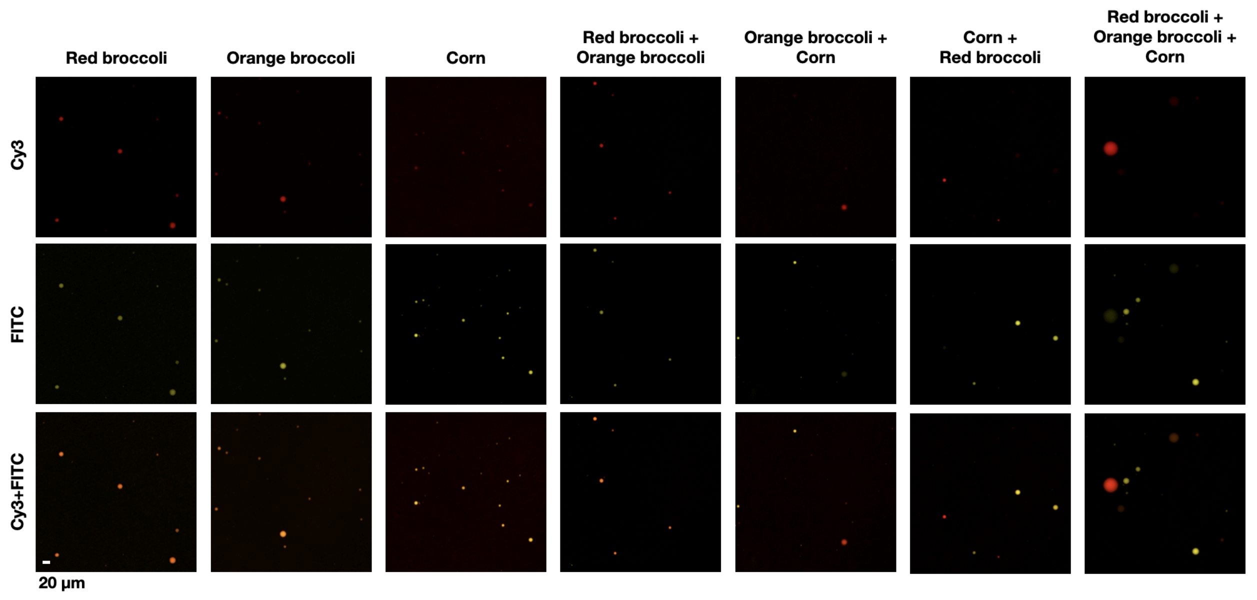

Supplementary Figure 8. Condensates with fluorogenic aptamers. RNA nanostars 4m3, 4m5, and 4m6 with Orange Broccoli, Corn, and Red Broccoli aptamers respectively, formed as single, double, and triple condensate systems in 40 mM HEPES, 100 mM KCl, 500 mM NaCl. Condestes were thermally treated by a quick 70°C melt followed by a 12h-long hold 50°C. Condensates were imaged using single filter channels Cy3 and FITC and showing the resulting overlay of filters Cy3 and FITC. Experiments were repeated three times, n=3; here we provide representative images.

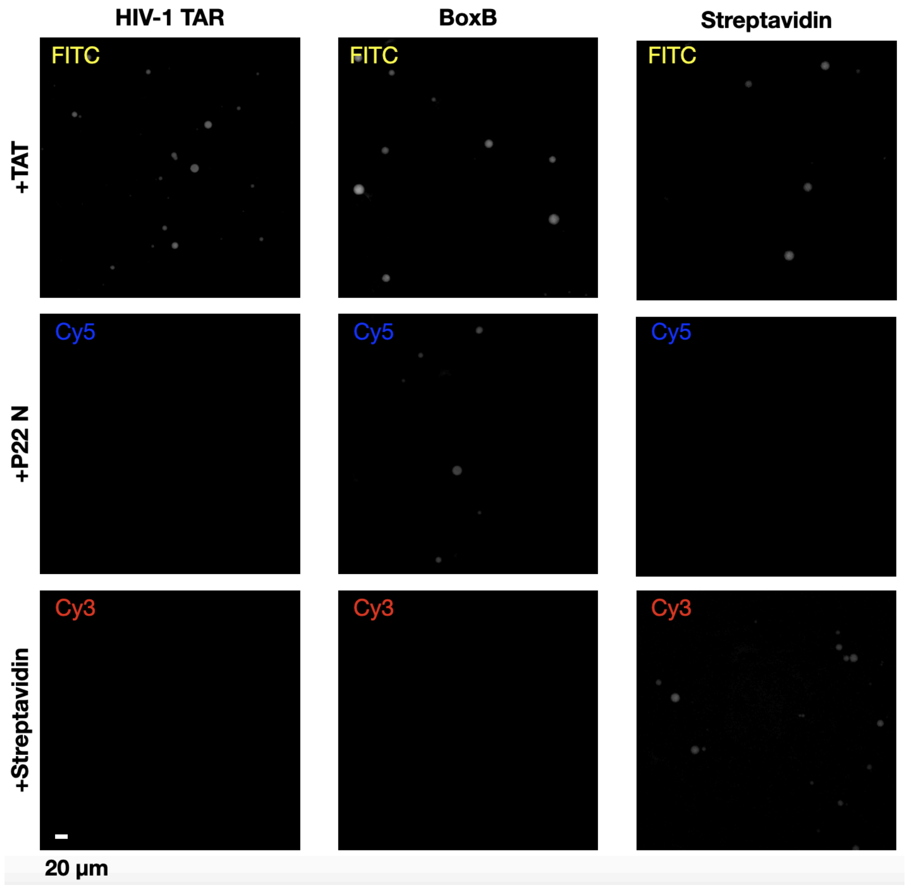

Supplementary Figure 9. Recruitment of peptides and protein of multistranded condensates. Fluorescence microscopy data of multistranded condensates 4m6 with TAR RNA, 4m5 with boxB RNA, and 4m3 with streptavidin binding aptamer in the presence of individual targets TAT peptide labeled with 6FAM, P22 N peptide labeled with AF647, and streptavidin protein labeled with AF555. Experiments were repeated three times, n=3; here we provide representative images. The positive charge of TAT peptide due to the rich arginine content causes indiscriminate binding to negatively charged RNA. Scale bar, 20 μm.

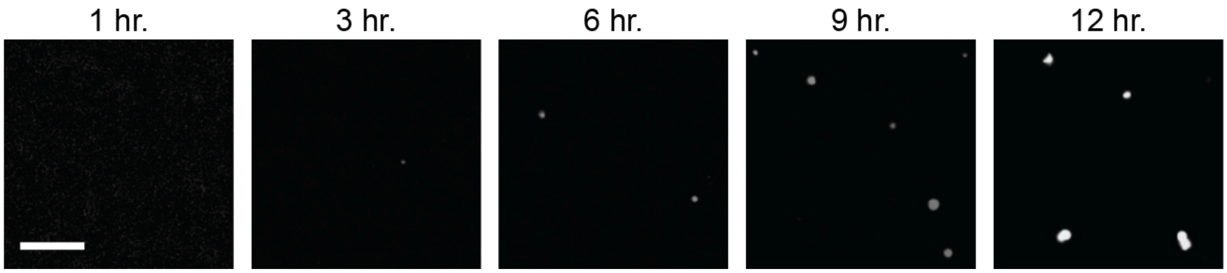

Supplementary Figure 10. Condensate growth during incubation at 50°C. We tracked the growth of our three-arm single-stranded nanostar 3sWT-stem1 during the hold step. The annealing mixture was aliquot into five samples, initiated simultaneously, and imaged at different time points. Condensates were stained with SYBR Gold and imaged. Images are enhanced for brightness and contrast with the same treatment for displaying. Experiments were repeated three times, n=3; here we provide representative imagess. Scale bar, 10 μm.

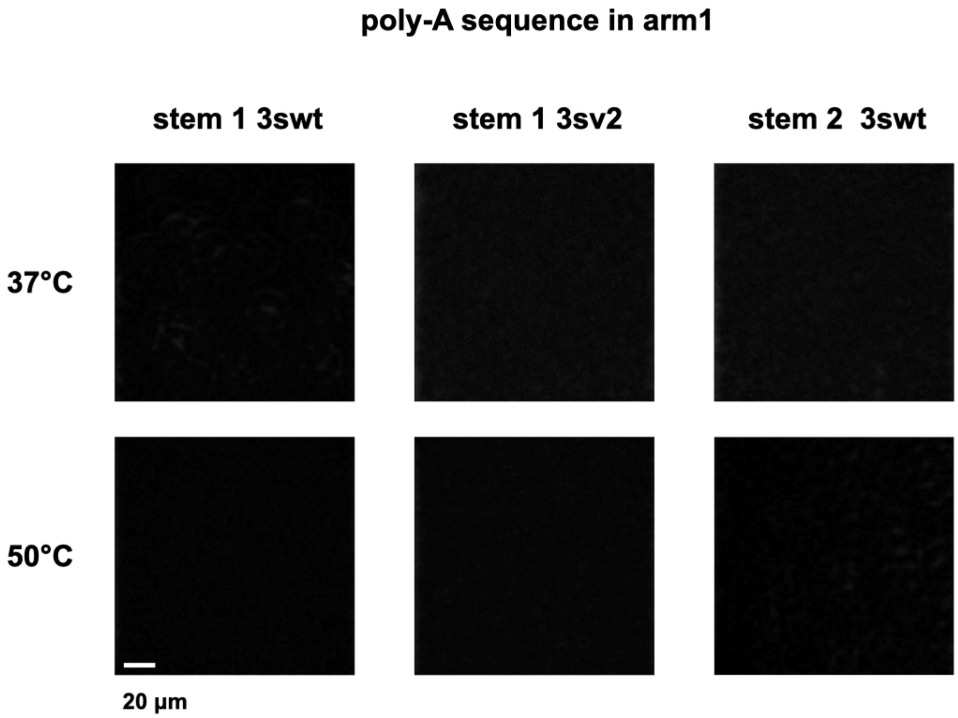

Supplementary Figure 11. Single-stranded nanostar variants with one of the three WT KLs replaced with the poly-A sequence formed no condensate. After the melt step, nanostars were incubated at 37°C or 50°C for 12h and cooled to 20°C before imaging. Samples were stained with SYBR Gold for imaging. Experiments were repeated three times, n=3; here we provide representative images. Scale bar, 20 μm.

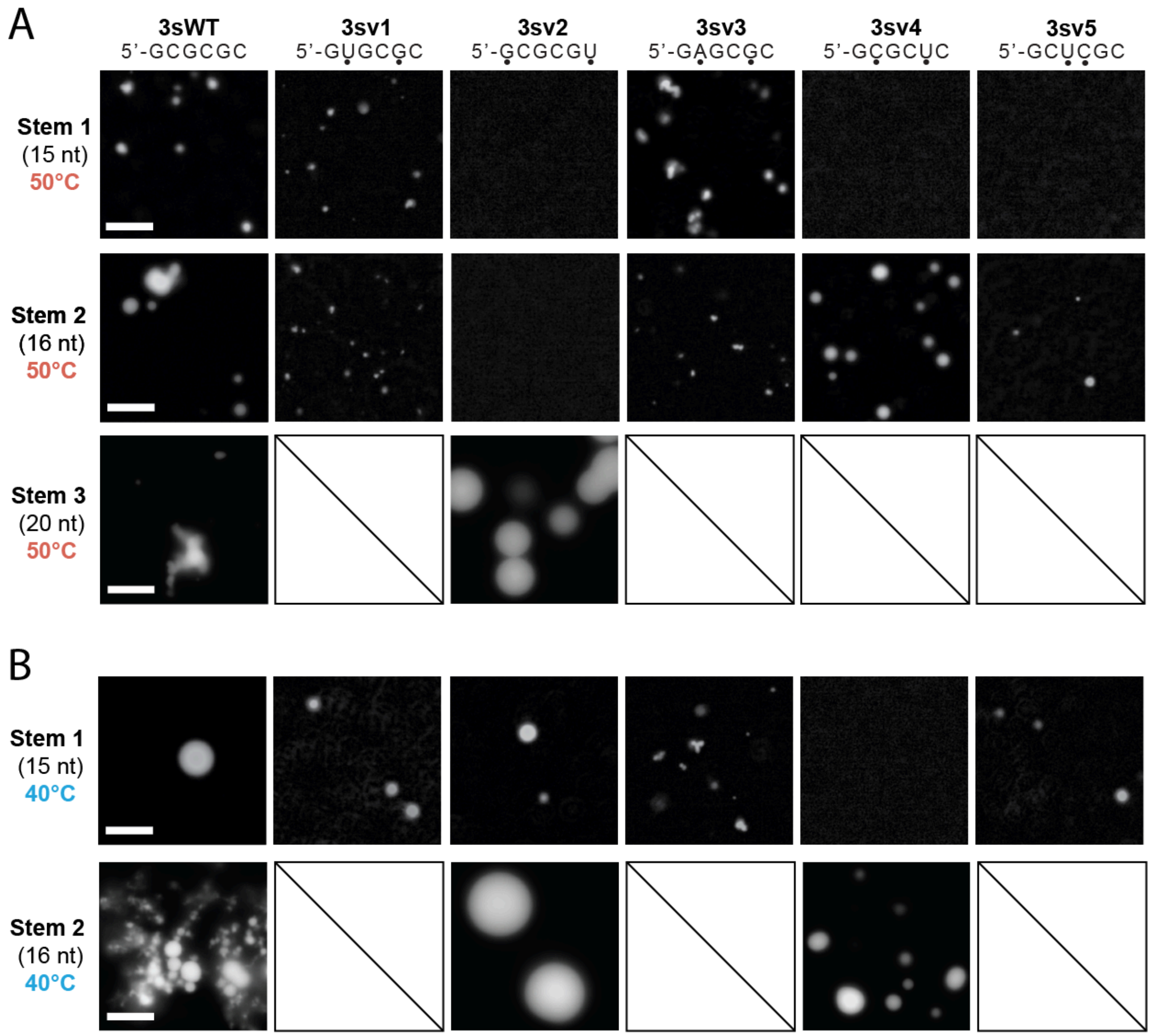

Supplementary Figure 12. Overview of fluorescence microscopy images for single nanostar designs. A) Condensates assembly from three types of stems and six types of self-complementary kissing loops that had no (3sWT) or two (all variants) mismatches (indicated by dots) with a 50°C hold. Stems have different lengths and sequences. **B)** Condensates assemble with a 40°C hold. All variants were assembled from purified RNA using our assembly buffer and the melt and hold annealing protocol (10 min at 70°C followed by 12 h hold at specified temperature), and stained with SYBR Gold for imaging. Experiments were done in n=3; here we provide representative images. Scale bar, 10 μm.

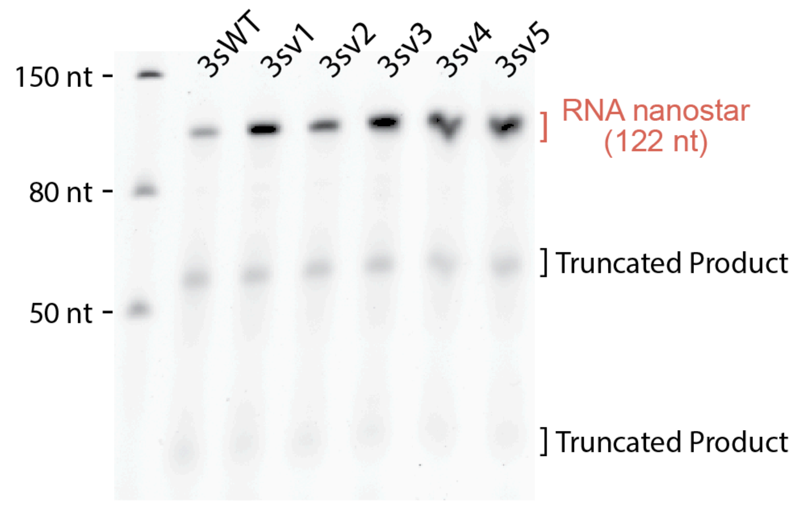

Supplementary Figure 13. Purified RNA Transcribed by T7 polymerase includes truncated product. In an 8% denaturing polyacrylamide gel, RNA purified from transcription products by T7 polymerase exhibited a major band corresponding to the desired stem 1 RNA nanostars, alongside two minor bands representing truncated products. The single-stranded regions of the DNA templates fold into nanostar structures, as predicted by NUPACK, while the formation of stem-loop structures causes early transcription termination due to polymerase dissociation. The gel was stained with 1x SYBR Gold. The gel was repeated at least five times with consistent results.

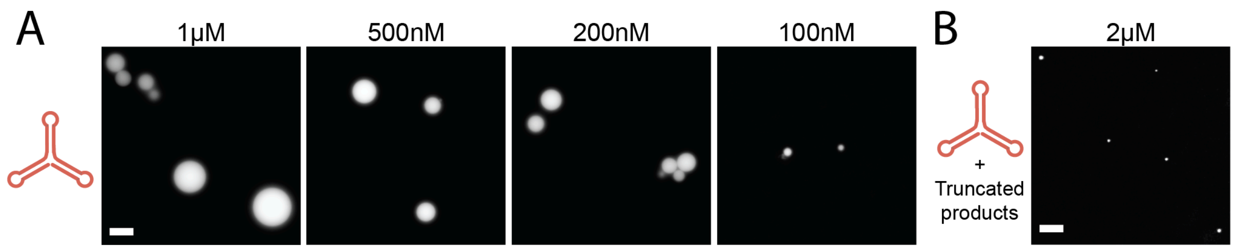

Supplementary Figure 14. The critical concentration for condensation is influenced by the presence of truncated transcription products. A) Variant 3sv2, stem 1 was transcribed, size-exclusion-column purified, and gel extracted. Condensates form at concentrations as low as 100 nM. **B)** Variant 3sv2, stem 1 was transcribed and size-exclusion-column purified, but not gel extracted. In the presence of truncated products, only tiny condensates can form at 2 μM. All samples were treated by the melt-and-hold protocol with a hold temperature at 40°C in our assembly buffer, and were stained with 1x SYBR Gold for imaging. Experiments were repeated three times, n=3; here we provide representative images. Scale bar, 10 μm.

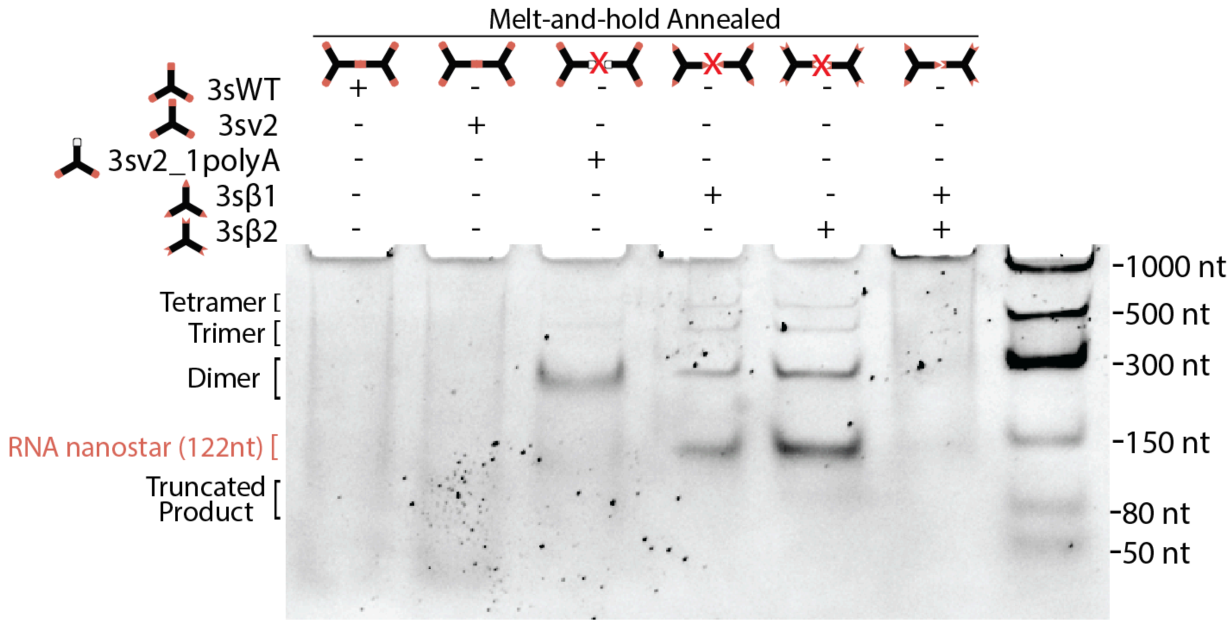

Supplementary Figure 15. Folding and interaction between single-stranded RNA treated with melt-and-hold protocol. In 8% native polyacrylamide gels, folded nanostars migrate slower than single-stranded RNA ladders. This characteristic is known for structured RNA. nanostars with palindromic kissing loops form complexes manifest as smears that cannot pass through the gel. Eliminating one kissing loop by changing it to poly A (5'-AAAAAA) dissociates the complex and generates predominantly dimers. The gel was stained with 1x SYBR Gold. The gel was repeated twice with consistent results.

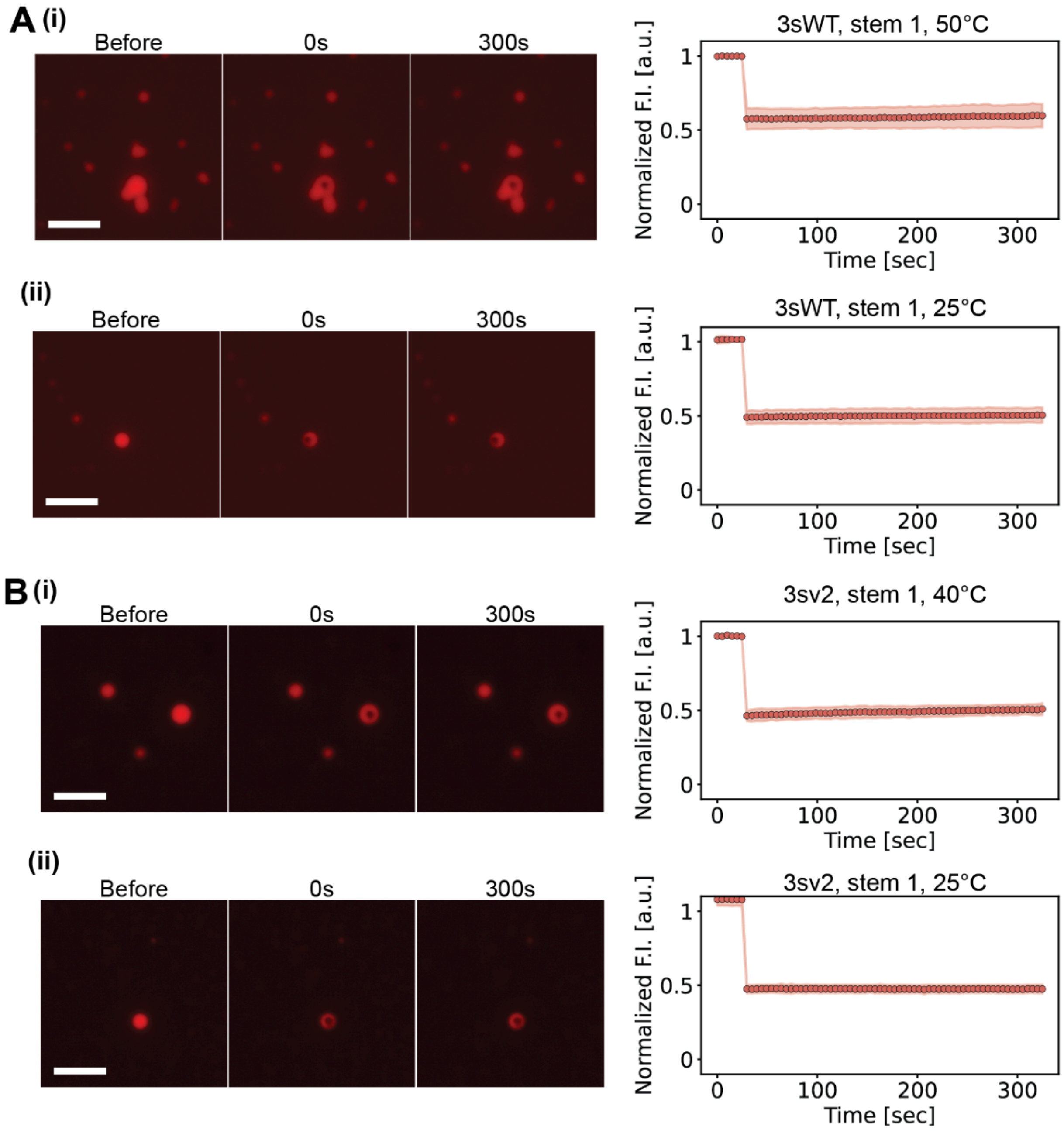

Supplementary Figure 16. Fluorescence recovery after photobleaching (FRAP) analysis of condensate formed from single-stranded nanostar treated with melt-and-hold protocol and co-transcribed showed little recovery. RNA was transcribed with 1% of CY3 tagged UTP and purified with size exclusion columns before imaging. Condensates were annealed with the heal-and-hold protocol and imaged at 50°C (**A (i)**, for variant 3sWT) or 40°C (**B(i)**, for variant 3sv2) or at room temperature (**A(ii) and B(ii)**). Orange dots indicate mean intensity at the corresponding time point. Shaded areas indicate standard error. Mean and standard error were calculated from 3 FOVs each belonging to a single replicate. Scale bar, 10 μm.

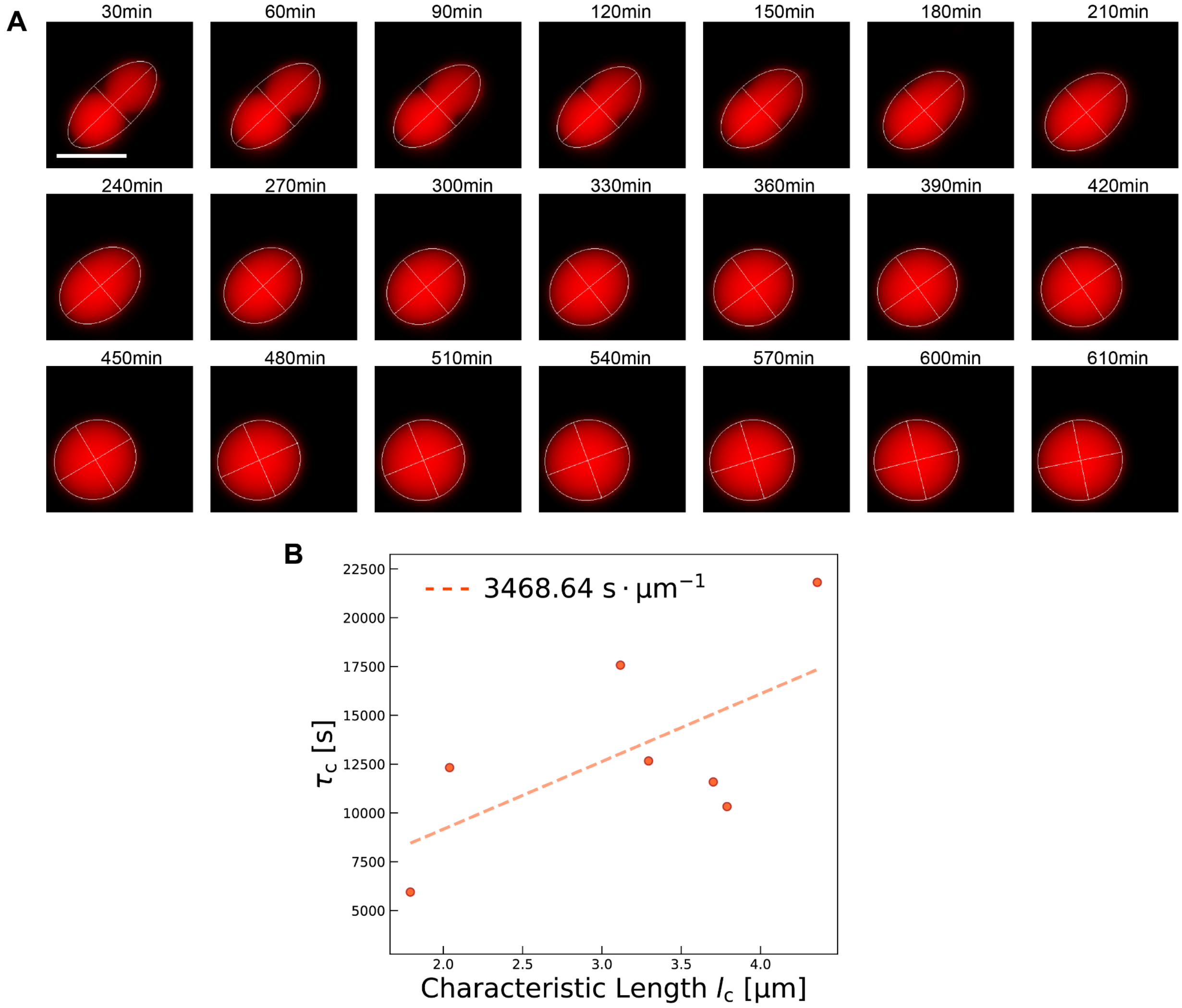

Supplementary Figure 17. Time-dependent coalescence of 3sv2 holding at 40 °C. (A) A coalescence event of 3sv2 droplets. **(B)** The time constant (*τc*) against the characteristic length (lc). Linear regression yields the inverse capillary velocity, μ/γ based on seven coalescence events from one sample. R^2 and p-value for the null hypothesis (H_0) of null slope are as follows: slope = 3468.64±1892.93, intercept: 2228.90±6195.51, R^2 = 0.63, p-value = 0.126. The characteristic length was calculated as the mean of two droplets at the beginning of a coalescence event. RNA was transcribed, column purified, and annealed following the melt-and-hold protocol. Condensates are stained with 1x SYBR Gold and imaged in a sealed chamber. Images are processed with SIFT for stack alignment to correct horizontal drifting. Data is extracted from one field of view from one experimental replicate. Scale bar, 10 μm.

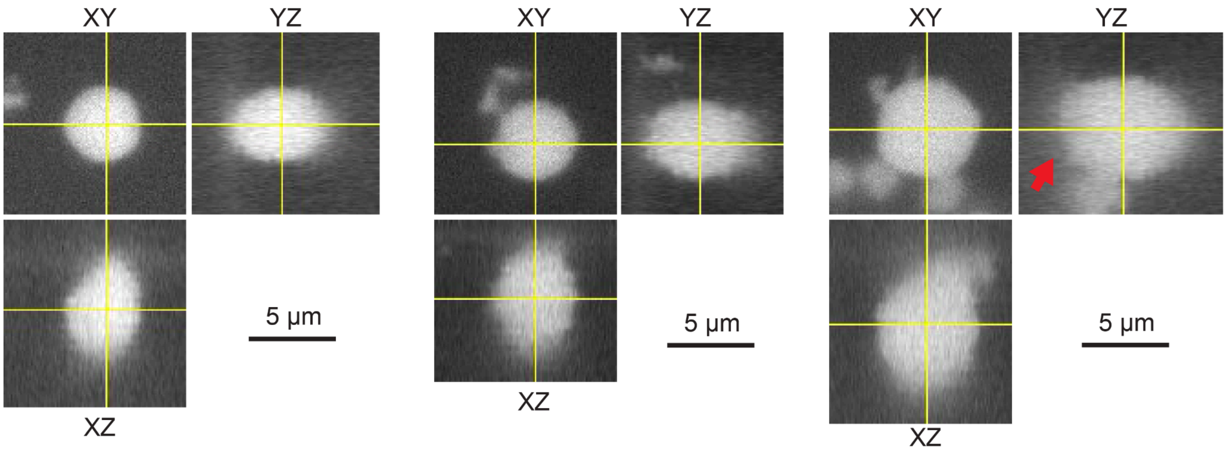

Supplementary Figure 18. Orthogonal views of confocal images of condensates demonstrate sedimentation of condensates on the glass slide surface with no significant deformation or wetting of the surface. Variant 3sv2, stem 1 was annealed following the melt-and-hold protocol by held at 40°C. The red arrow indicated one droplet where slight wetting of the surface was observed. Samples are stained with 1x SYBR Gold. The extension of droplets on the z-axis direction could be attributed to photonic leakage of confocal imaging. Data is extracted from three fields of view from one sample. Scale bar, 5 μm.

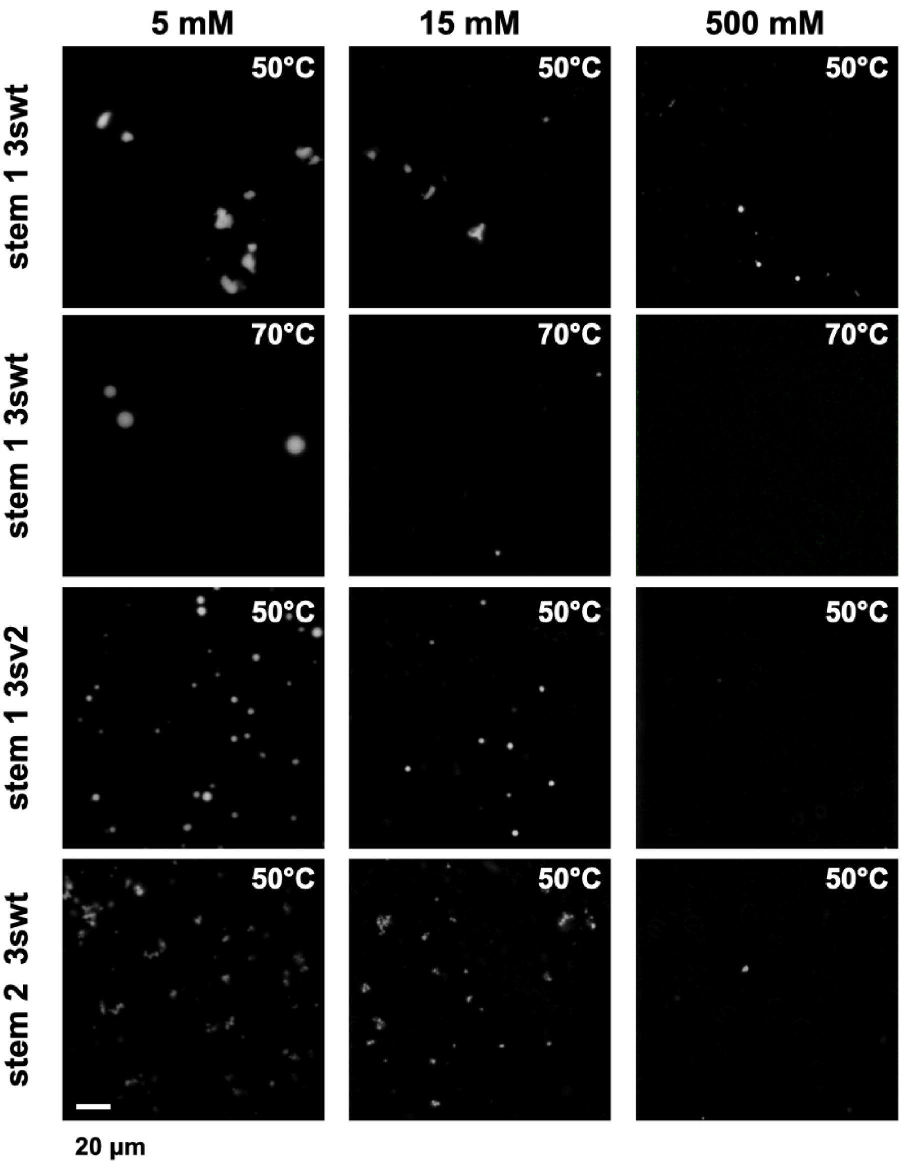

Supplementary Figure 19. Condensate formation at different magnesium conditions. Fluorescence micrographs showing condensate morphology of three motifs in the presence of 40 mM HEPES and a variable MgCl2 concentration. Condensates were annealed at 50°C for 12h and cooled to 20°C before imaging. Samples were stained with SYBR Gold for imaging. Experiments were repeated three times, n=3; here we provide representative images. Scale bar, 20 µm.

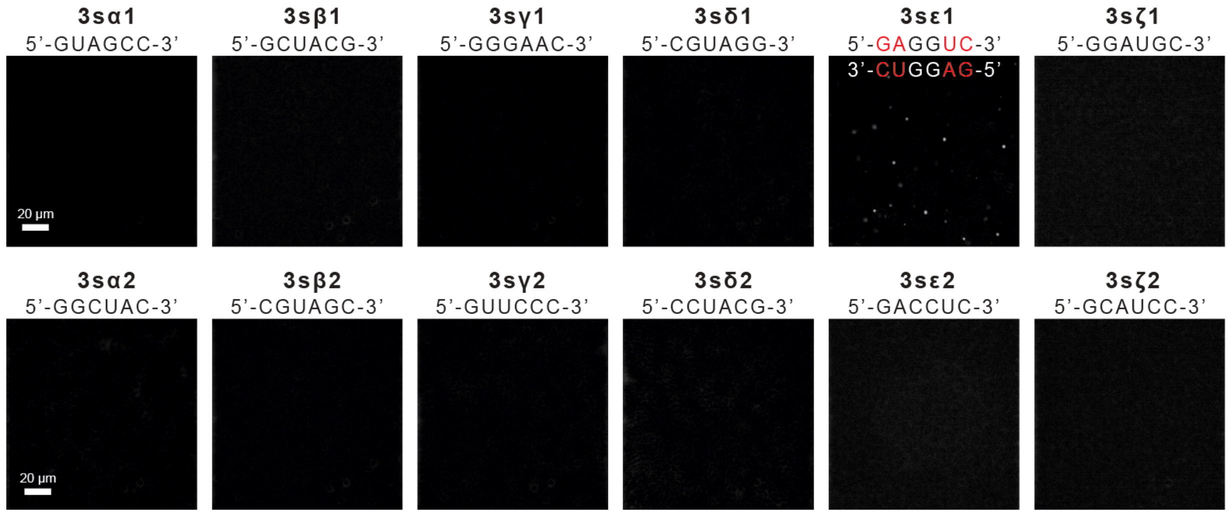

Supplementary Figure 20. Condensates do not form when annealing individual components of the two nanostar designs. Motifs have identical stems, and these results indicate that stem-stem interactions do not determine condensate formation. Only variant 3sε1 form condensates; the KL of this variant includes two guanine pairs surrounded by a total of four complementary base pairs, confirming that mismatches within kissing loops can be tolerated and still yield condensates. However, condensation is sensitive to the type of substitution and to the position of mismatches within kissing loops. Experiments were repeated three times, n=3; here we provide representative images. Scale bar, 20 µm.

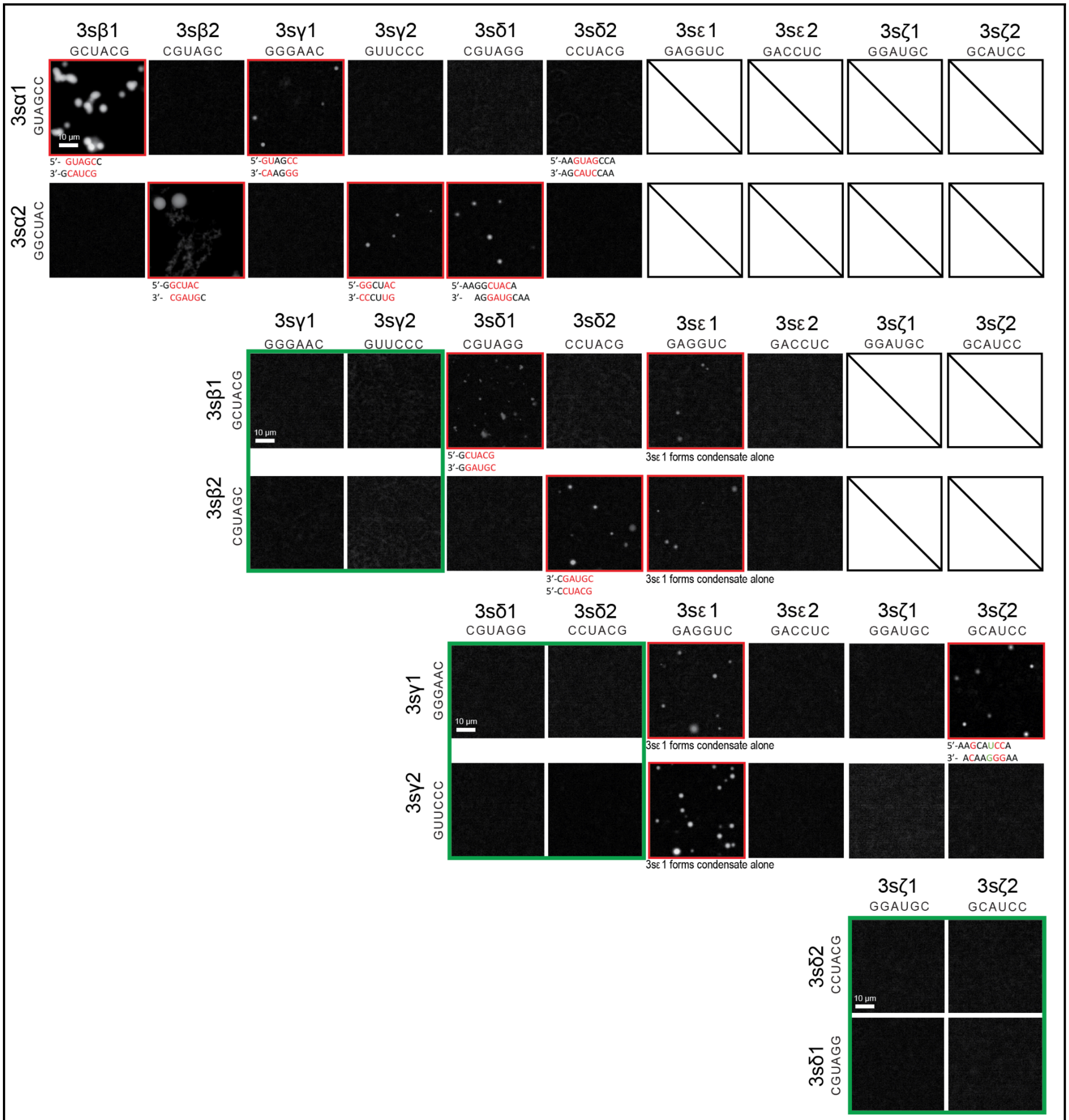

Supplementary Figure 21. Cross orthogonality between two nanostar condensates. Each box includes representative microscopy images of samples prepared by annealing (melt and hold at 50°C) two nanostars that are part of different nanostar-generating condensates. The name and sequence are marked to the left of each row and at the top of each column. Pairs that produce condensates are highlighted in red. Possible sequence base pairings are highlighted in red underneath images. Pairs that are fully orthogonal are in green. Pair 3sβ and 3sγ are used for orthogonality demonstrations in Fig. 4C. Pair 3sζ fails as it generates gel-like networks which do not match our goal of generating liquid-like droplets (see Fig. 3H in the manuscript). Pair 3sε fails as nanostar 3sε1 yields condensates on its own (Supplementary Figure 20). Experiments were repeated three times, n=3; here we provide representative images. Scale bar, 10 µm.

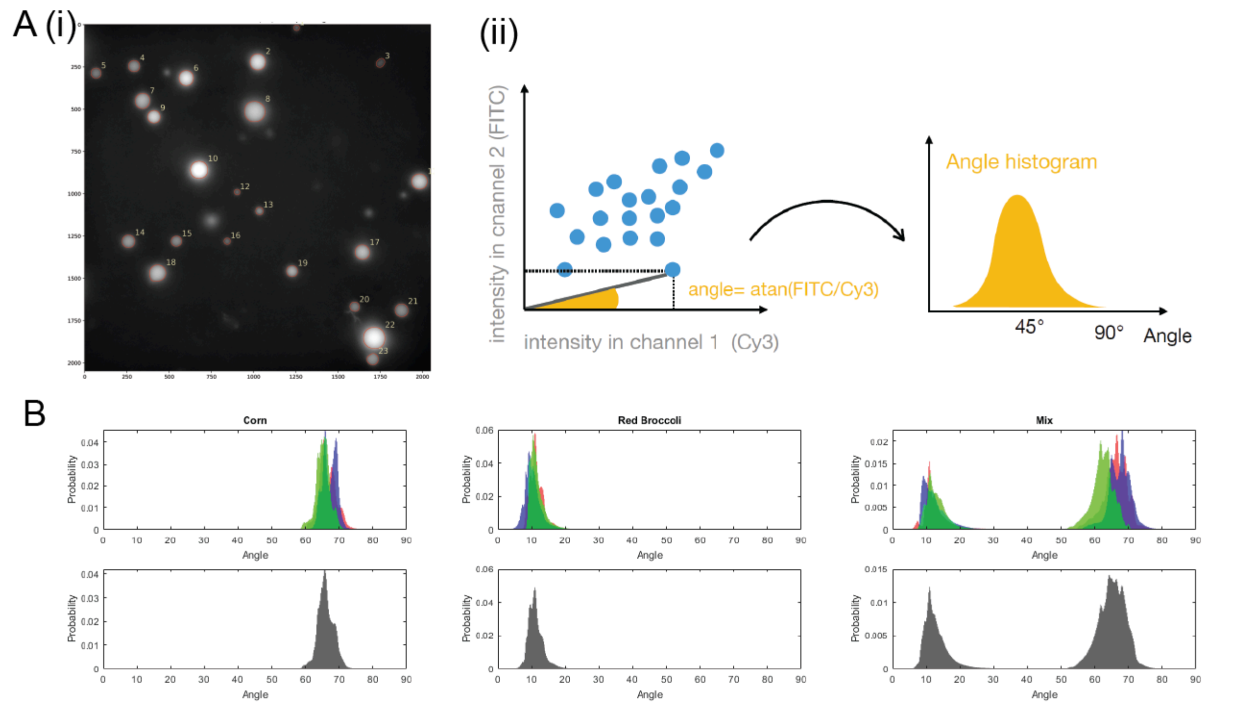

Supplementary Figure 22. Coordinate angle mapping workflow and results for each sample. (**A**) (i) Example of an overlay of fluorescent image and labeled regions that were drawn from corresponding binary mask. We extracted pixel intensities in the CY3 and FITC channels within these labeled regions (circled in red). (ii) For each pixel, a coordinate angle was calculated as arctan (FITC_intensity/CY3_intensity). A histogram of coordinate angles was generated for each sample (FOV = 10). (**B**) Histograms of n = 3 samples (top panel, in red, green, and blue) and sum of the three (bottom panel, in gray) for 3sγ_Corn, 3sβ_Red Broccoli, and one-pot annealing of the two. The skewing towards smaller angles (for Corn) and bigger angles (for Red Broccoli) were due to saturated pixels in the FITC channel (for Corn) or CY3 channel (for Red Broccoli). Total RNA concentration doubled in mixed annealing, resulting in bigger droplets and more saturation, thus increasing the skewing of peaks towards the middle.

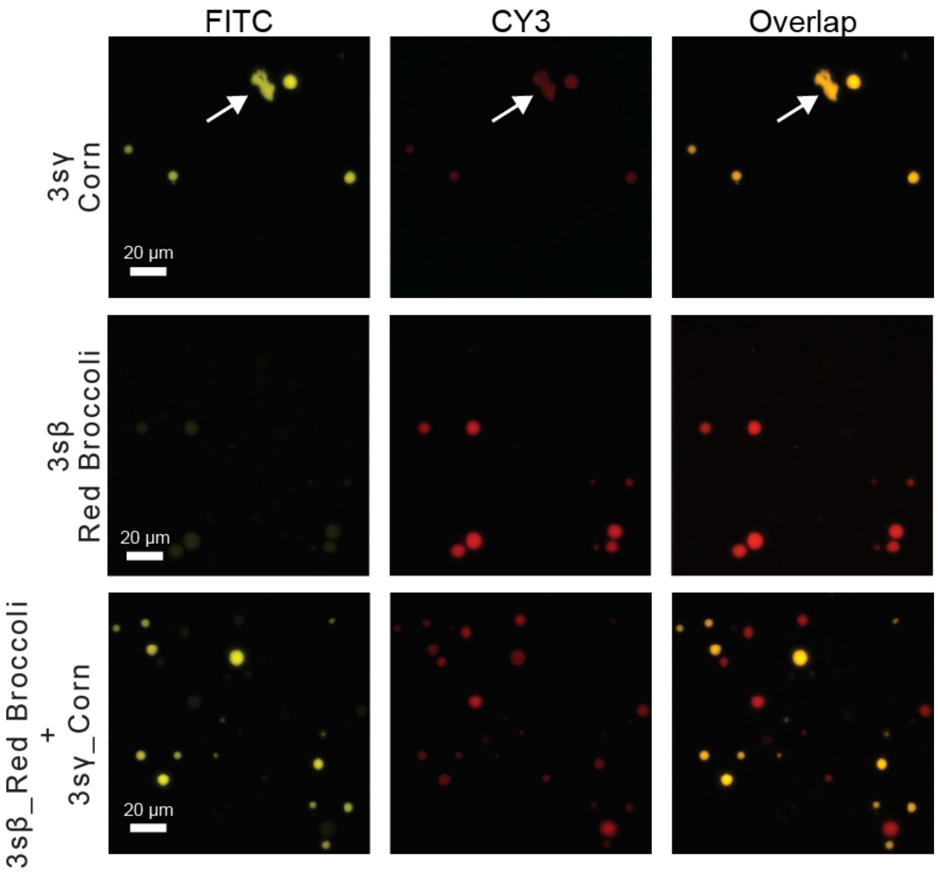

Supplementary Figure 23. Example images of individually and simultaneously annealed two nanostar condensates that include fluorogenic aptamers. Rows 1 and 2 from the top are individually annealed nanostars, row 3 shows example images of simultaneously annealed nanostars. The nanostars include 25% of aptamer-appended strands. White arrows point at non-spherical condensates whose formation may be driven by the dimerization of Corn aptamers. Experiments were repeated three times, n=3; images are representative examples.

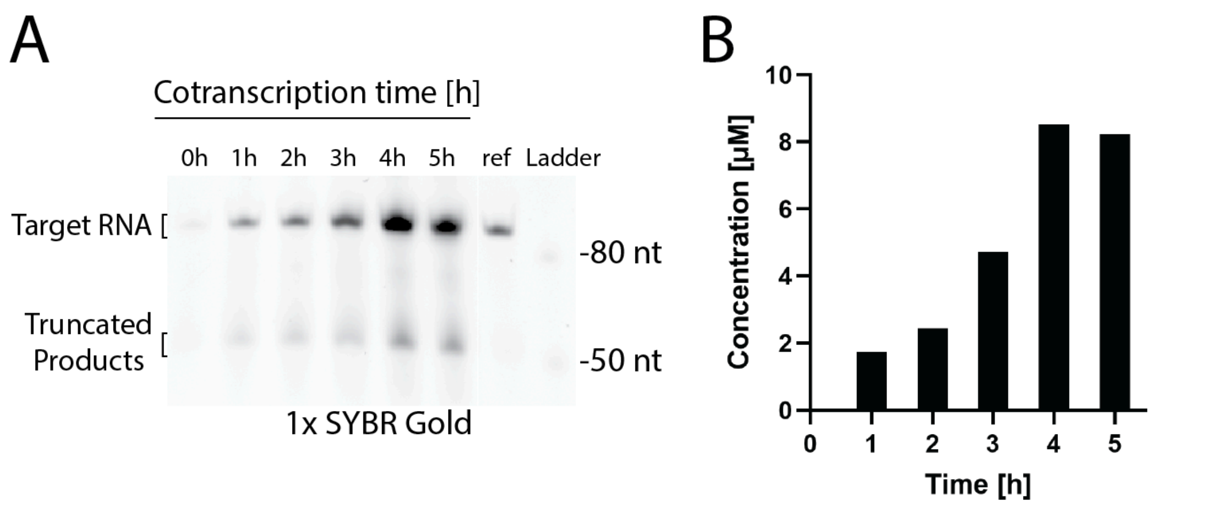

Supplementary Figure 24. Estimation of RNA co-transcription rate. A) 8% Denaturing PAGE gel; the reference strand (3sv2, gel extracted) is alongside products of transcription, terminated after 5 hours by adding DNase I. Transcription protocol is consistent with the one used to produce condensates (10nM DNA template, high-yield transcription buffer). The concentration of reference strands was measured using a Nanodrop2000c spectrophotometer as absorption at 260 nm and calculated following the method outlined in Section 1.2.2. Transcription samples were diluted 25 times with water, and 2μL of each sample was loaded. Reference strands were loaded at concentrations of 100 nM in the well (left, totaling 0.2 pmol). Gel electrophoresis for this experiment was performed once. **B)** The co-transcription rate of RNA was calculated based on data from panel A (n=1). The total pixel intensity of bands corresponding to the target product (122 nt) was extracted and calibrated to the concentration of the reference strand.

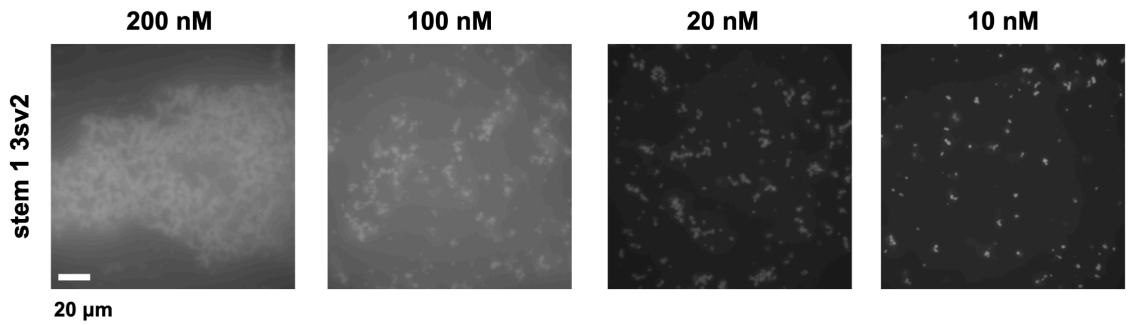

Supplementary Figure 25. DNA template titration for co-transcription experiments. Fluorescence micrographs at 2h timepoint showing co-transcriptional condensate formation with a variable final DNA template concentration. Experiments were repeated three times, n=3; images are representative examples. Scale bar, 20 μm.

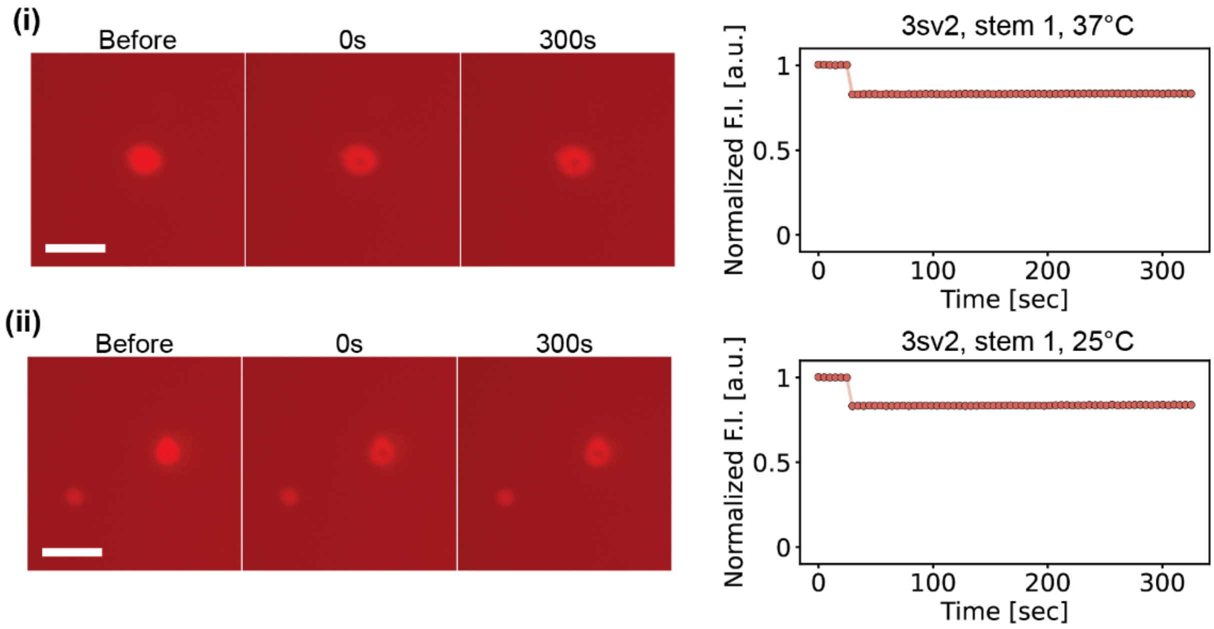

Supplementary Figure 26. Fluorescence recovery after photobleaching (FRAP) analysis of condensate formed from co-transcription of single-stranded nanostar showed little recovery. Condensates were co-transcribed with 1% CY3-labeled UTP, diluted 10 times with 1x co-transcription buffer to reduce background fluorescence, and imaged at 37°C **(i)** or at room temperature **(ii)**. Orange dots indicate mean intensity at the corresponding time point. Mean and standard error were calculated from 3 FOVs each belonging to one experimental replicate; we obtained a remarkably low standard error, which confirms the lack of recovery.

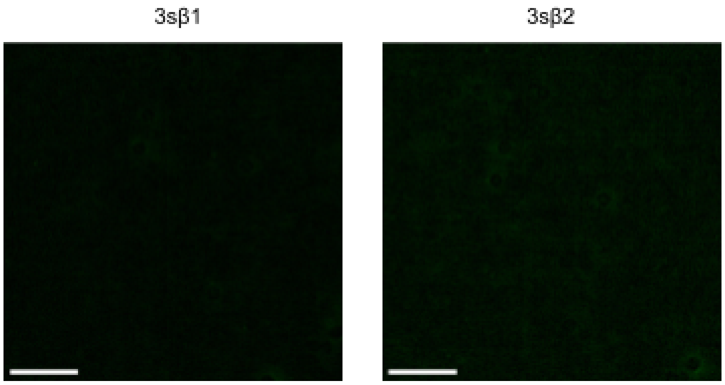

Supplementary Figure 27. Individual nanostars with non-palindromic KL cannot form condensates co-transcriptionally. Fluorescence micrographs at 1h timepoint showing no co-transcriptional condensate formation for either nanostar when transcribed alone. Experiments were repeated three times, n=3; images are representative examples. Scale bar, 10 μm.

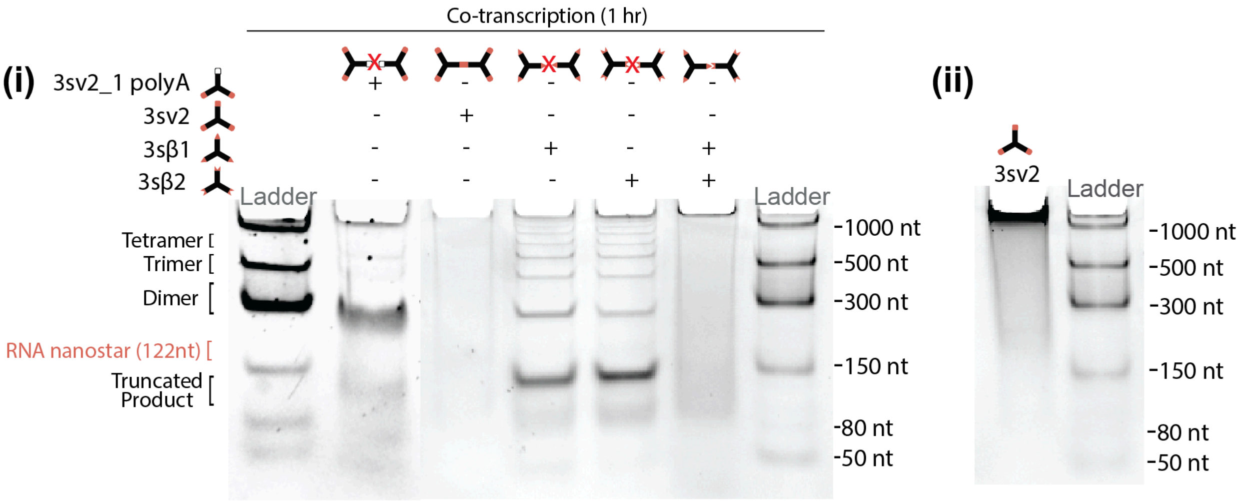

Supplementary Figure 28. Co-transcriptional folding and interaction between single-stranded RNA. (i) In 8% native polyacrylamide gels, nanostars with palindromic kissing loops form complexes that manifest as smears that cannot pass through the gel. Eliminating one kissing loop by changing it to poly A (5'-AAAAAA) dissociates the complex and generates predominantly dimers. **(ii)** Increased loading of 3sv2 demonstrates apparent retention of RNA in wells. The gel was stained with 1x SYBR Gold. The gel was repeated twice with consistent results.

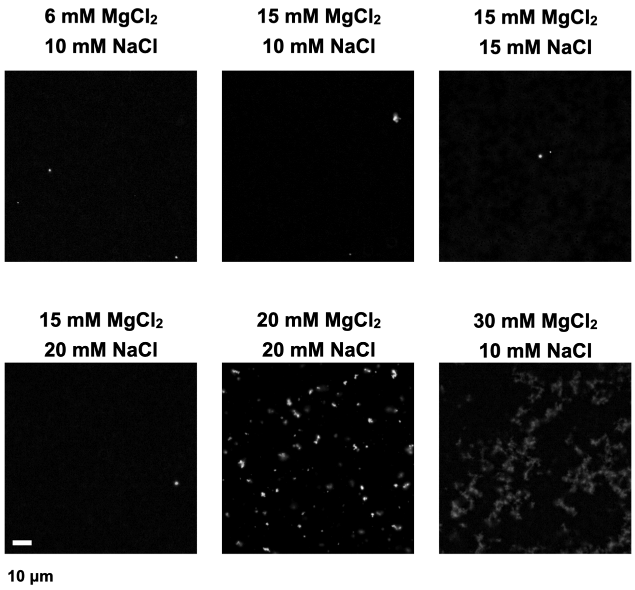

Supplementary Figure 29. Magnesium and sodium titration for co-transcription experiments of the two-nanostar system (3sβ**).** Fluorescence micrographs at 1h timepoint showing co-transcriptional condensate formation with various $MgCl_2$ and NaCl concentrations. Each template is added at 10 nM. Reaction conditions are consistent with those listed in section 1.3.2. Experiments were repeated three times, n=3; images are representative examples. Scale bar, 10 µm.

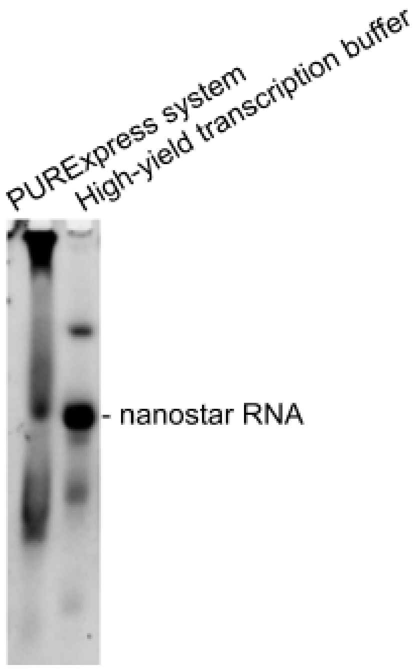

Supplementary Figure 30. Nanostar RNA (stem 1 3sv2) transcribed with the PURExpress system or the high-yield transcription buffer. In 8% native polyacrylamide gels, nanostars that were transcribed from the PurExpress system (left lane) had more byproducts than the ones that were transcribed with the high-yield transcription buffer (right lane). DNase I was used to stop the transcription after 3 hours of incubation at 37°C. The 8% denaturing polyacrylamide gel was stained with 1x SYBR Gold. The gel was repeated twice with consistent results.

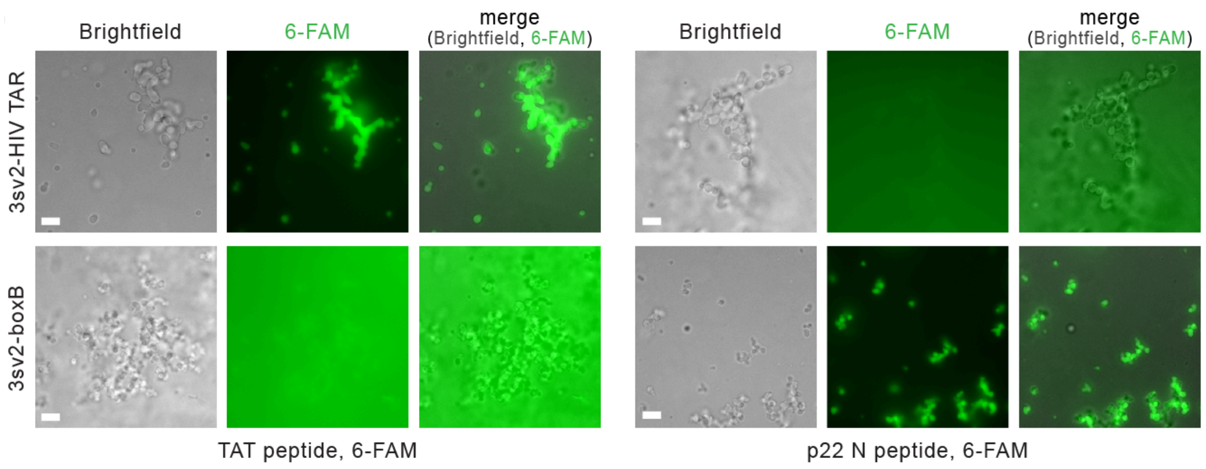

Supplementary Figure 31. Adding peptides after transcription. 3sv2 nanostars were modified to include TAR (top) or boxB (bottom) aptamer, recruiting either TAT or p22 N peptide labeled with 6-FAM. No significant difference between condensate growth was observed compared with adding peptide at the beginning of transcription (Fig. 5G), indicating minimal interference of peptide on condensation. Experiments were repeated three times, n=3; images are representative examples. Scale bars: 10 µm.

3 Supplementary Tables

5' SE sequence duplex	ΔG (kcal/mol)
GC	-2.12
GCGC	-8.17
GUAC	-4.5
GAGCUC	-11.43
GUAUAC	-6.96
GCUAGC	-11.17

Supplementary Table 1. NUPACK ΔG calculations of sticky-end duplexes at 25°C using rna95 (NUPACK3) parameters, some dangles (NUPACK3) ensemble, and 1M Na+.

Supplementary References

1. Zadeh, J. N. *et al.* NUPACK: Analysis and design of nucleic acid systems. *J. Comput. Chem.* **32**, 170–173 (2011).
2. Filonov, G. S., Song, W. & Jaffrey, S. R. Spectral Tuning by a Single Nucleotide Controls the Fluorescence Properties of a Fluorogenic Aptamer. *Biochemistry* **58**, 1560–1564 (2019).
3. Song, W. *et al.* Imaging RNA polymerase III transcription using a photostable RNA-fluorophore complex. *Nat. Chem. Biol.* **13**, 1187–1194 (2017).
4. Tan, R. & Frankel, A. D. Structural variety of arginine-rich RNA-binding peptides. *Proc. Natl. Acad. Sci. U. S. A.* **92**, 5282–5286 (1995).
5. Yamamoto, R. *et al.* A novel RNA motif that binds efficiently and specifically to the Ttat protein of HIV and inhibits the trans-activation by Tat of transcription in vitro and in vivo. *Genes Cells* **5**, 371–388 (2000).
6. Leppek, K. & Stoecklin, G. An optimized streptavidin-binding RNA aptamer for purification of ribonucleoprotein complexes identifies novel ARE-binding proteins. *Nucleic Acids Res.* **42**, e13 (2014).
7. Sato, Y., Sakamoto, T. & Takinoue, M. Sequence-based engineering of dynamic functions of micrometer-sized DNA droplets. *Sci Adv* **6**, eaba3471 (2020).
8. Clever, J. L., Wong, M. L. & Parslow, T. G. Requirements for kissing-loop-mediated dimerization of human immunodeficiency virus RNA. *J. Virol.* **70**, 5902–5908 (1996).
9. Warner, K. D. *et al.* A homodimer interface without base pairs in an RNA mimic of red fluorescent protein. *Nat. Chem. Biol.* **13**, 1195–1201 (2017).
