# Supplementary material for: Modular RNA motifs for orthogonal phase separated compartments
Source: Nat Commun. 2024 Jul 30;15:6244. doi: 10.1038/s41467-024-50003-x (PMC11289419; doi:10.1038/s41467-024-50003-x)
Supplement: Supplementary file 3 — Description of Additional Supplementary Files [file 41467_2024_50003_MOESM3_ESM.pdf]

Description of Additional Supplementary Files

Supplementary Data 1 - Includes a list of oligonucleotide sequences used in this study.
