# Supplementary figures and images for: Modular RNA motifs for orthogonal phase separated compartments
Source: Nat Commun. 2024 Jul 30;15:6244. doi: 10.1038/s41467-024-50003-x (PMC11289419; doi:10.1038/s41467-024-50003-x)

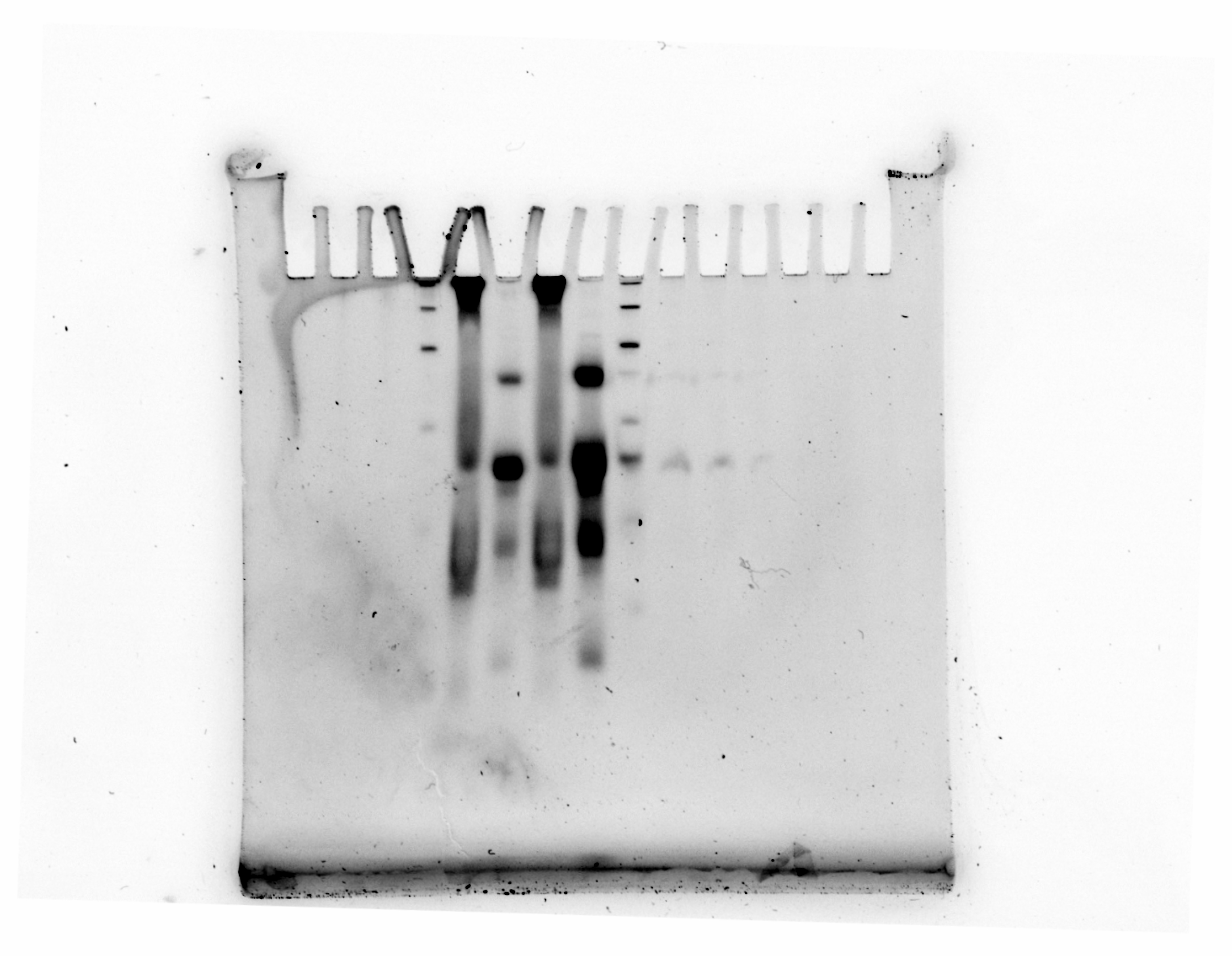

Supplement: Supplementary file 6 — Source data [file 41467_2024_50003_MOESM6_ESM.zip › Source data files/Uncropped gel images/Gel - Supplementary Fig. 30.jpg]

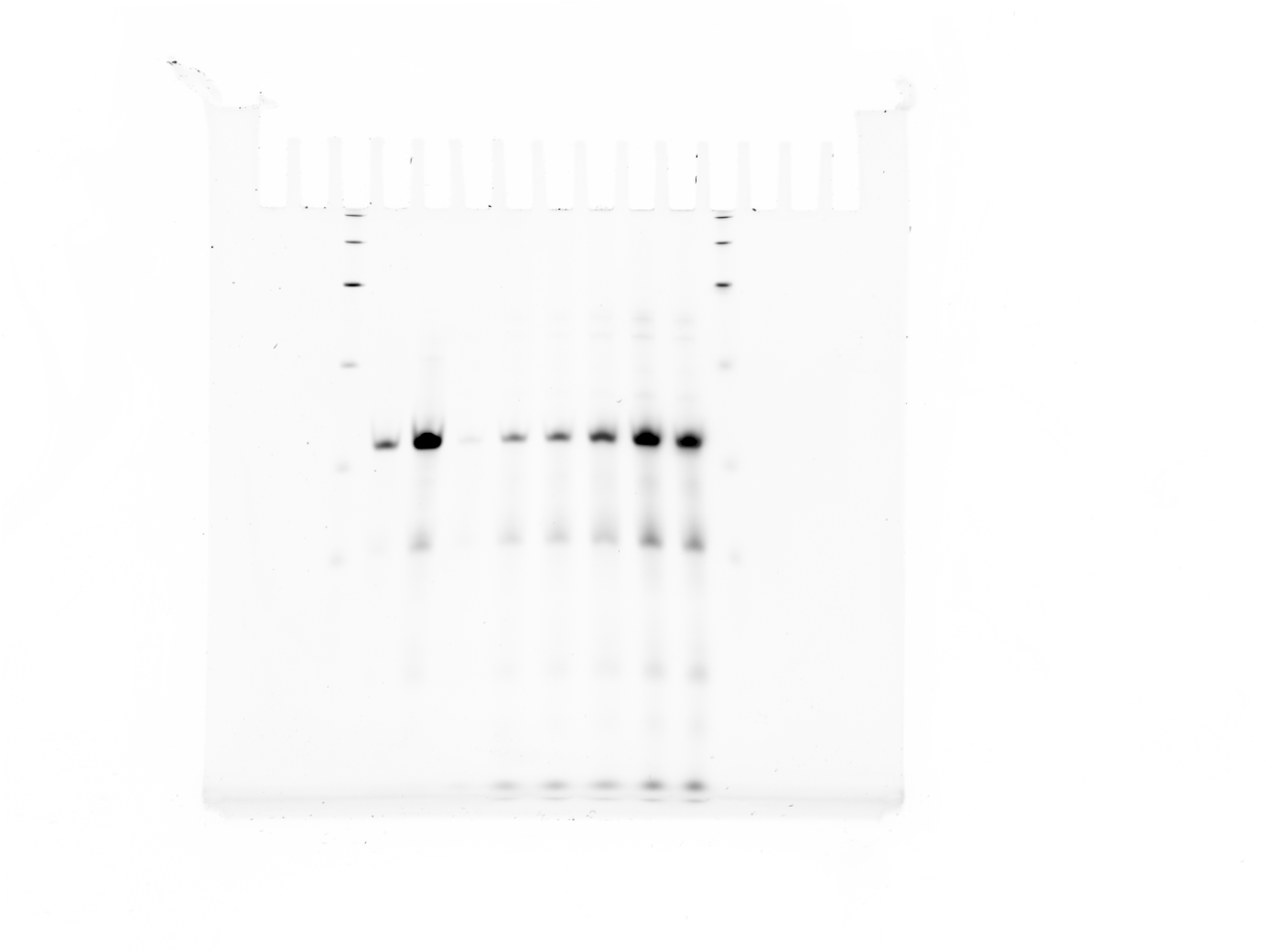

Supplement: Supplementary file 6 — Source data [file 41467_2024_50003_MOESM6_ESM.zip › Source data files/Uncropped gel images/Gel - Supplementary Fig. 24.tif]

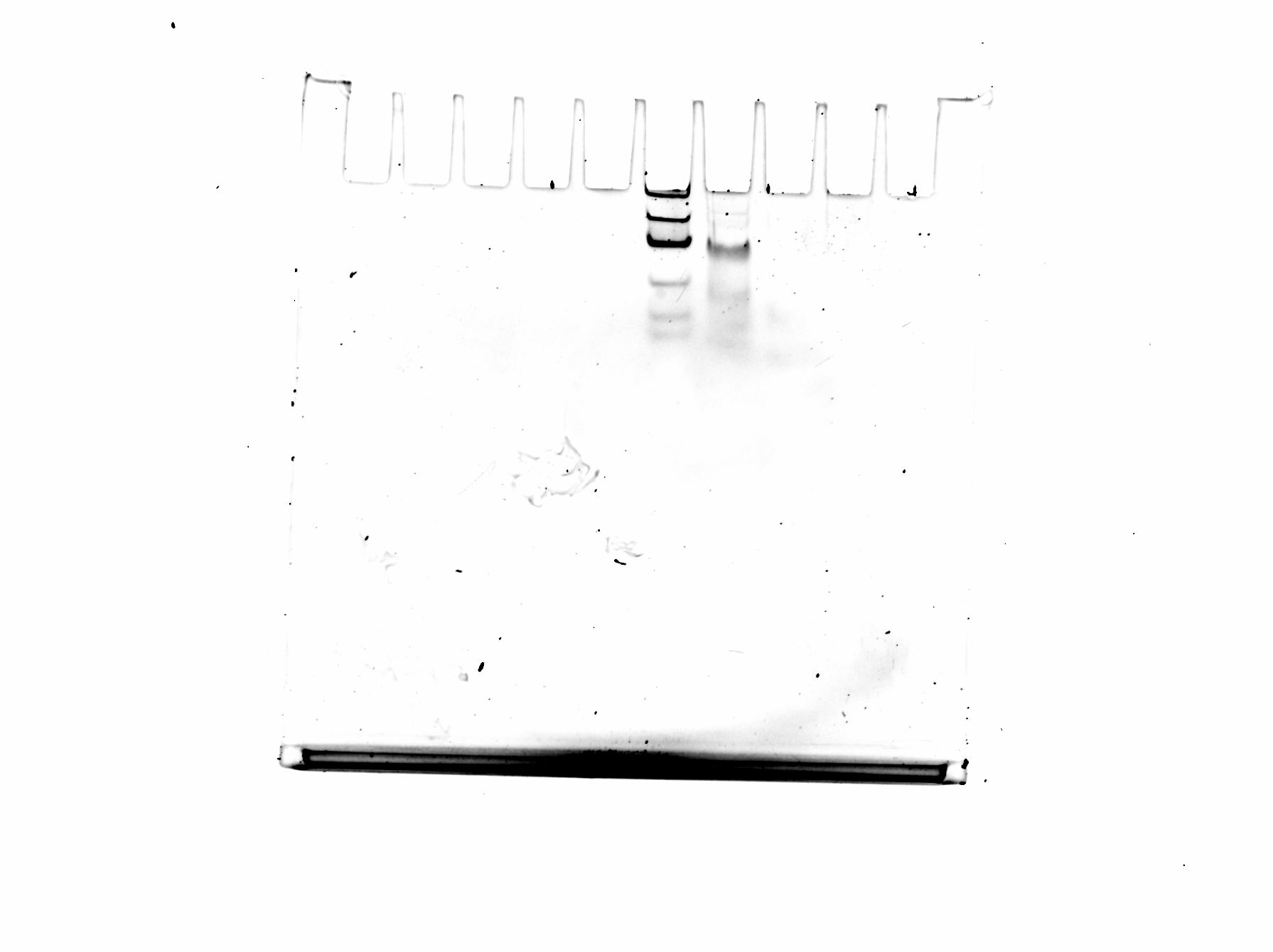

Supplement: Supplementary file 6 — Source data [file 41467_2024_50003_MOESM6_ESM.zip › Source data files/Uncropped gel images/Gel - Supplementary Fig. 28 -1.tif]

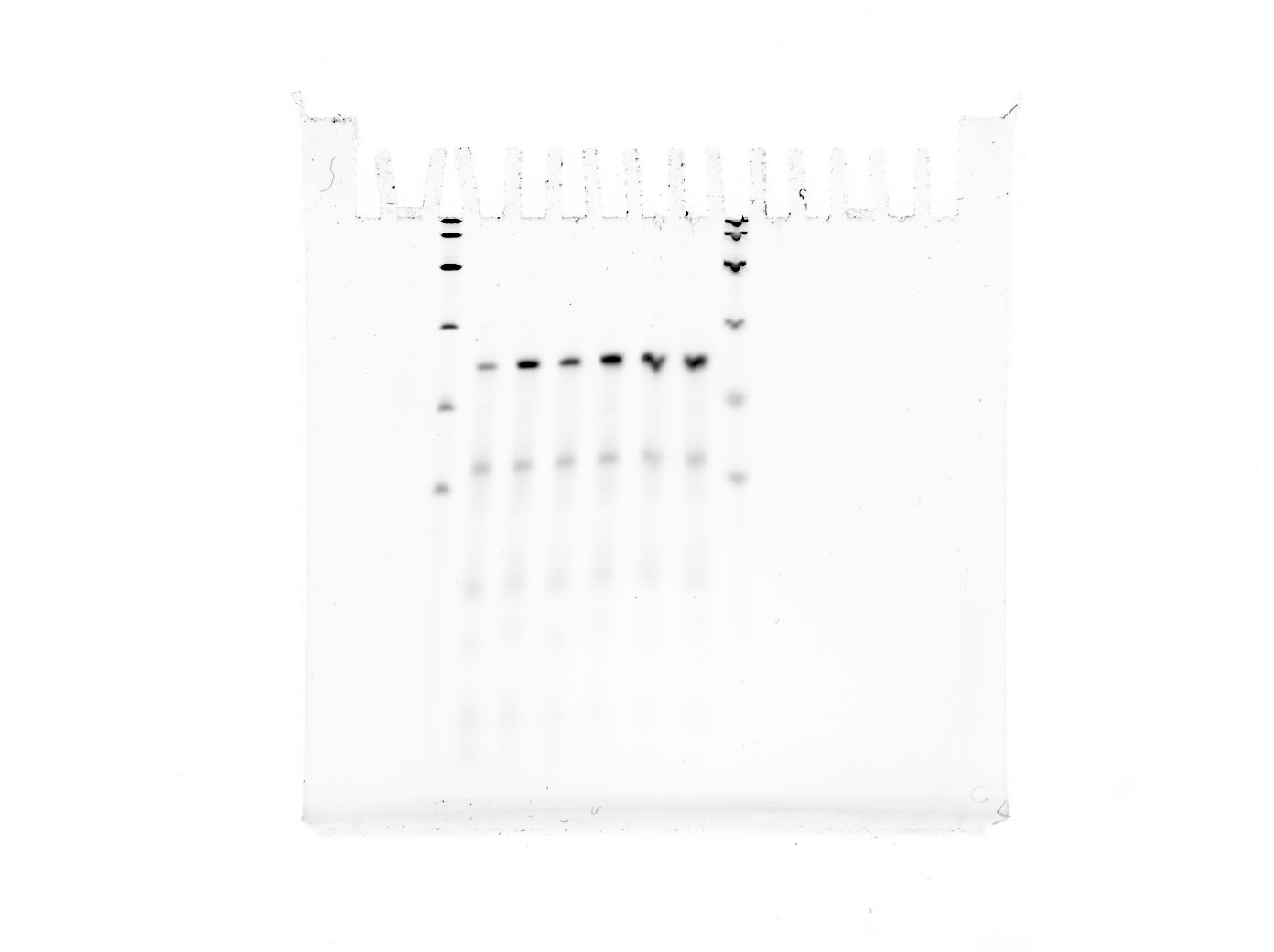

Supplement: Supplementary file 6 — Source data [file 41467_2024_50003_MOESM6_ESM.zip › Source data files/Uncropped gel images/Gel - Supplementary Fig. 13.tif]

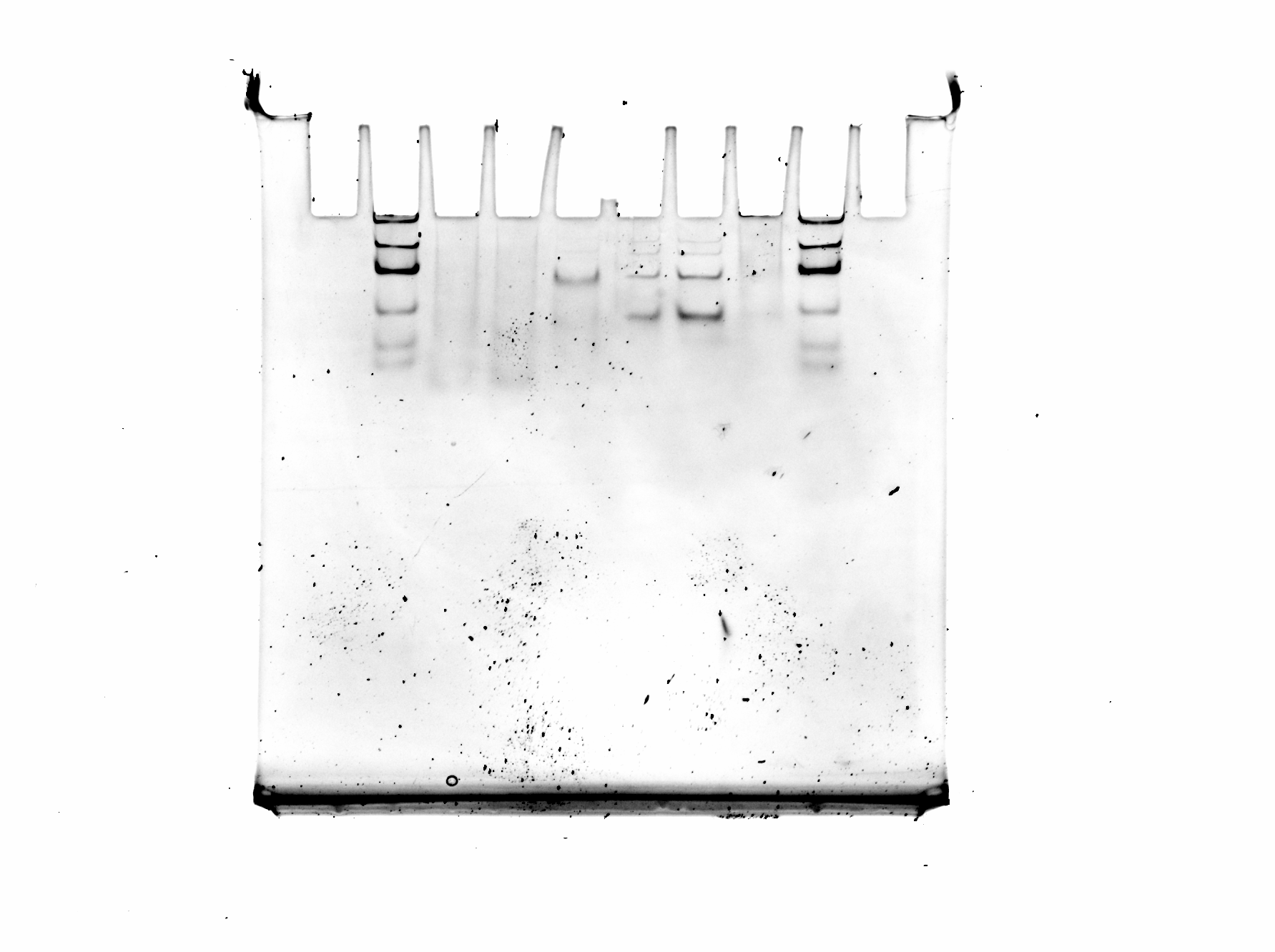

Supplement: Supplementary file 6 — Source data [file 41467_2024_50003_MOESM6_ESM.zip › Source data files/Uncropped gel images/Gel - Supplementary Fig. 15.tif]

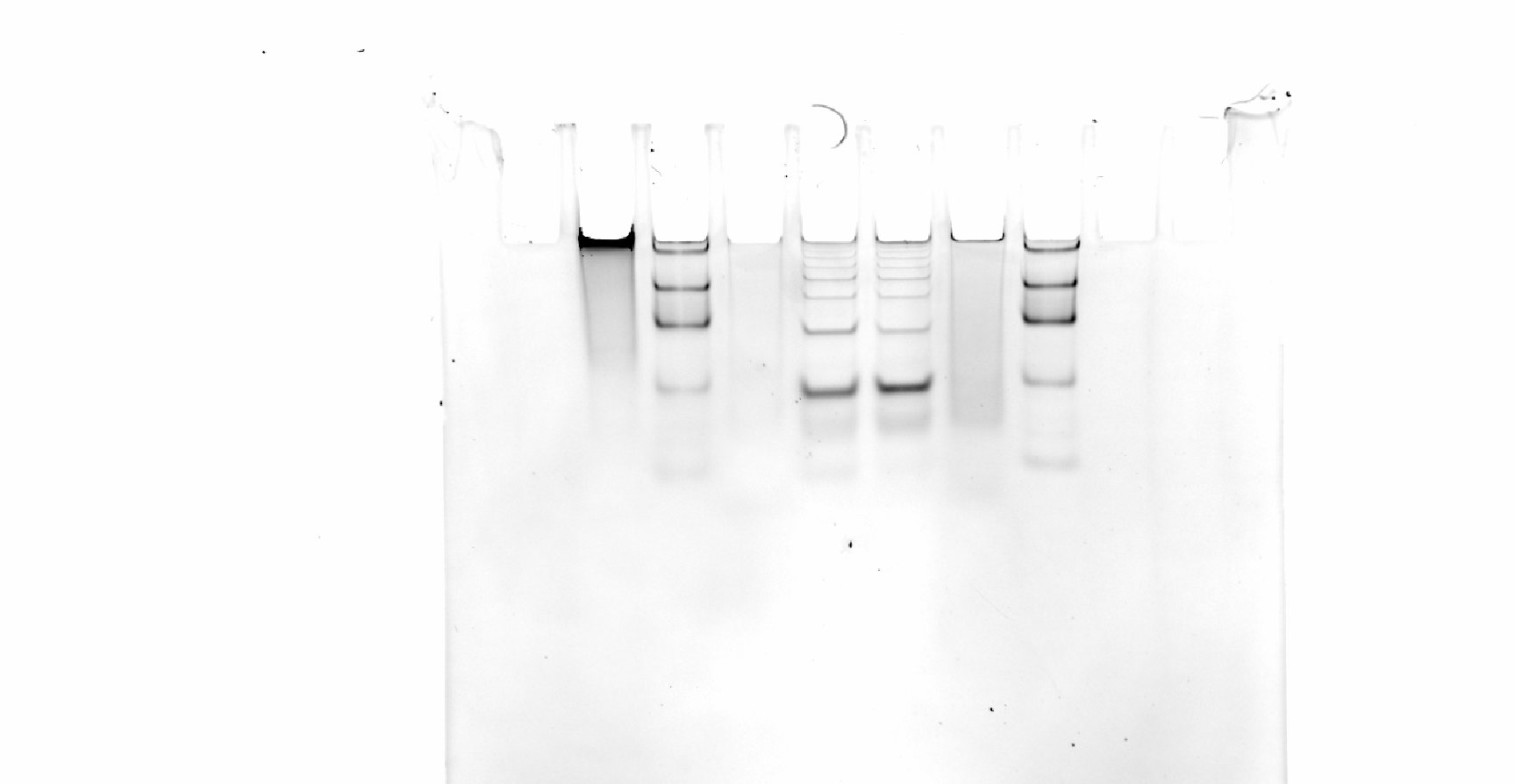

Supplement: Supplementary file 6 — Source data [file 41467_2024_50003_MOESM6_ESM.zip › Source data files/Uncropped gel images/Gel - Supplementary Fig. 28-2.tif]

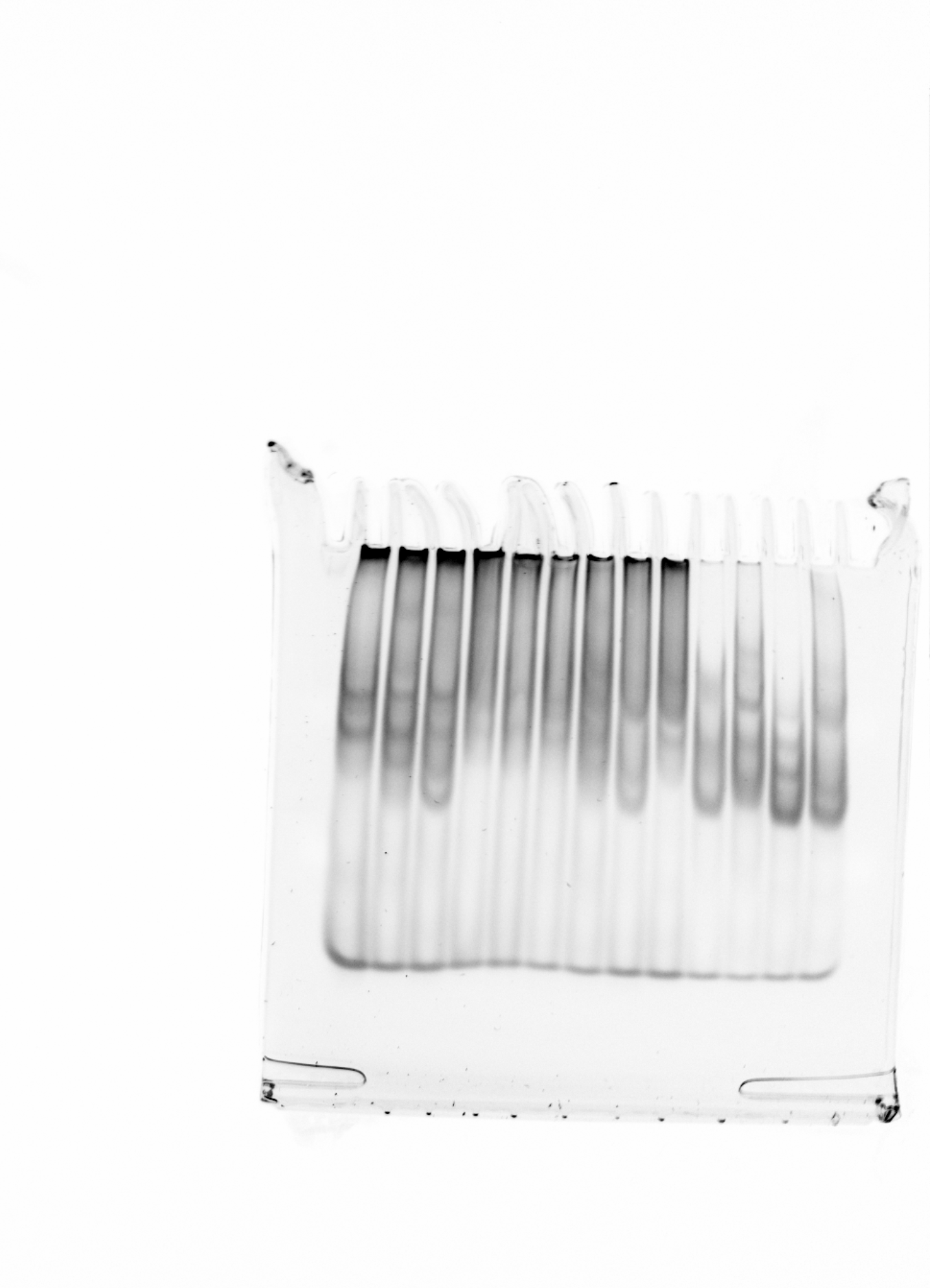

Supplement: Supplementary file 6 — Source data [file 41467_2024_50003_MOESM6_ESM.zip › Source data files/Uncropped gel images/Gel - Supplementary Fig. 5.tif]

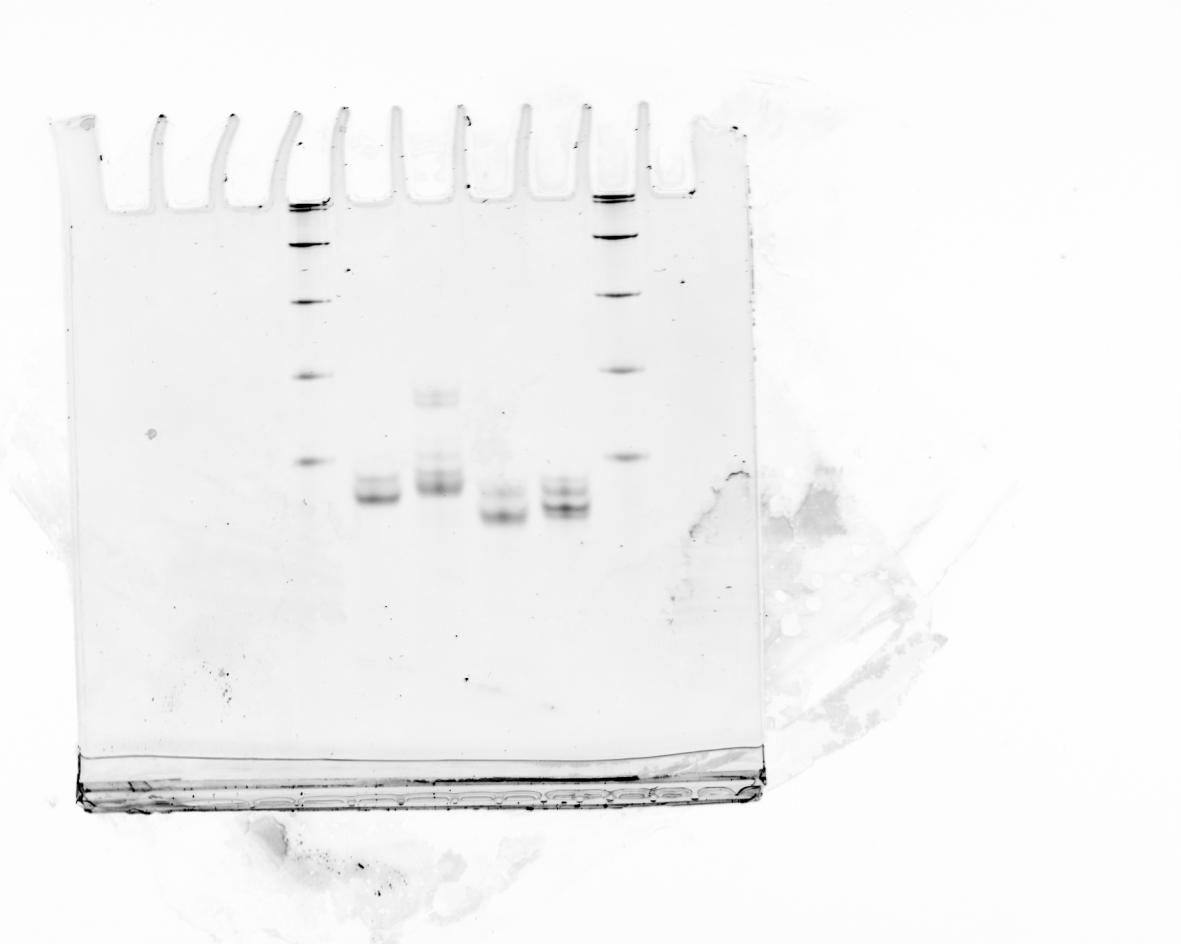

Supplement: Supplementary file 6 — Source data [file 41467_2024_50003_MOESM6_ESM.zip › Source data files/Uncropped gel images/Gel - Supplementary Fig. 4.tif]
